# Twenty-Five Years of Human Urinary Biomonitoring of Mycotoxins: Analytical Advances and Applications in Risk Assessment

**DOI:** 10.3390/toxins18090390

**Published:** 2026-09-09

**Authors:** Oana Mîrza, Denisia Pașca, Lara Manyes, Anamaria Cozma-Petruț, Roxana Banc, Simona Codruța Hegheș, Lorena Filip, Béla Kiss

**Affiliations:** 1 Department of Bromatology, Hygiene, Nutrition, Faculty of Pharmacy, “Iuliu Hațieganu” University of Medicine and Pharmacy Cluj-Napoca, 6 Louis Pasteur, 400349 Cluj-Napoca, Romania; oana.stanciu@umfcluj.ro (O.M.); anamaria.cozma@umfcluj.ro (A.C.-P.); roxana.banc@umfcluj.ro (R.B.); lfilip@umfcluj.ro (L.F.); 2 Laboratory of Toxicology, Institute of Forensic Medicine Cluj-Napoca, 3-5 Clinicilor, 400006 Cluj-Napoca, Romania; kbela@umfcluj.ro; 3 Faculty of Nursing and Health Sciences, “Iuliu Hațieganu” University of Medicine and Pharmacy Cluj-Napoca, 6 Louis Pasteur, 400349 Cluj-Napoca, Romania; 4 Biotech AgriFood Lab, Faculty of Pharmacy and Food Sciences, University of Valencia, Burjassot, 46100 Valencia, Spain; 5 Department of Toxicology, Faculty of Pharmacy, “Iuliu Haţieganu” University of Medicine and Pharmacy Cluj-Napoca, 6 Louis Pasteur, 400349 Cluj-Napoca, Romania; 6 Department of Drug Analysis, Faculty of Pharmacy, “luliu Hatieganu” University of Medicine and Pharmacy, 6 Louis Pasteur Street, 400349 Cluj-Napoca, Romania; cmaier@umfcluj.ro; 7 Academy of Romanian Scientists (AOSR), 3 Ilfov St, 050044 Bucharest, Romania

**Keywords:** mycotoxins, human exposure, biological fluids, biomonitoring, risk assessment

## Abstract

Mycotoxins are secondary fungal metabolites that have continuously attracted research interest, both for their potential negative effects on animal and human health and for the economic losses they entail. Studies assessing exposure to mycotoxins have become increasingly widespread. The aim of this study was to provide a comprehensive description of mycotoxins and to evaluate the evolution of research on monitoring mycotoxin exposure using human urine samples, concerning analytical techniques and occurrence. Studies published in English over the last 25 years from four databases (Web of Science, PubMed, Science Direct, Scopus) were analyzed. Evaluating 73 original articles, a constant interest in the analysis of ochratoxin A in human urine samples could be observed (maximum level 148 ng/mL), a fact also justified by its demonstrated toxicity. The most commonly used analytical technique to evaluate mycotoxin presence in human urine was liquid chromatography coupled with high-performance detectors. The limitations of the review include the large period covered and the lack of mycotoxin concentrations expressed in ng biomarker/mL urine in some articles. Finally, an overview of how mycotoxin levels in human urine can be applied to mycotoxin exposure risk assessment was included.

## 1. Introduction

Mycotoxins are a heterogeneous group of chemical compounds known as toxic secondary metabolites (metabolites that are not necessary for the fungus’s regular growth and reproduction) produced by a variety of filamentous fungi such as *Fusarium*, *Aspergillus*, *Penicillium* and *Alternaria* species, but also by *Claviceps*, *Mucor*, *Trichoderma*, *Trichothecium*, *Myrothecium*, *Pyrenophora*, *Stachybotrys*, *Cladosporium*, and *Helminthosporium* species [1,2]. Modern studies on mycotoxins began in 1960, after the discovery and characterization of aflatoxins (AFs); since then, more than 400 compounds have been included in this class, but only about 50 of them have been studied in more detail in terms of occurrence and toxicity [3,4]. The scientific classification of mycotoxins is according to the origin genera, but it must be considered that different fungal species can produce the same mycotoxin and one fungus can produce various mycotoxins at the same time. Therefore, mycotoxins are commonly classified according to their chemical and toxicological similarities [5,6,7,8]. Thus, a short classification of the main mycotoxins comprises: AFs—aflatoxin B1 (AFB1) and aflatoxin M1 (AFM1) being the most studied; ochratoxins, with the most potent toxin ochratoxin A (OTA); zearalenone (ZEA); fumonisins (FUMOs), with fumonisin B1 (FB1) being the most dangerous; trichothecenes, mainly deoxynivalenol (DON), T-2 toxin (T-2) and HT-2 toxin (HT-2); patulin (PAT), and ergot alkaloids [9,10,11,12]. In addition, other mycotoxins have also been identified and described, such as citrinin (CIT), α-cyclopiazonic acid (CPA), various emerging mycotoxins including enniatins (ENs), beauvericin (BEA), nivalenol (NIV), diacetoxyscirpenol (DAS), fusaric acid, moniliformin (MON), sterigmatocystin (STG), or different *Alternaria* mycotoxins, with alternariol, alternariol monomethyl ether, tenuazonic acid, altertoxins and tentoxin being the most representative [12,13,14].

**Figure 1:**
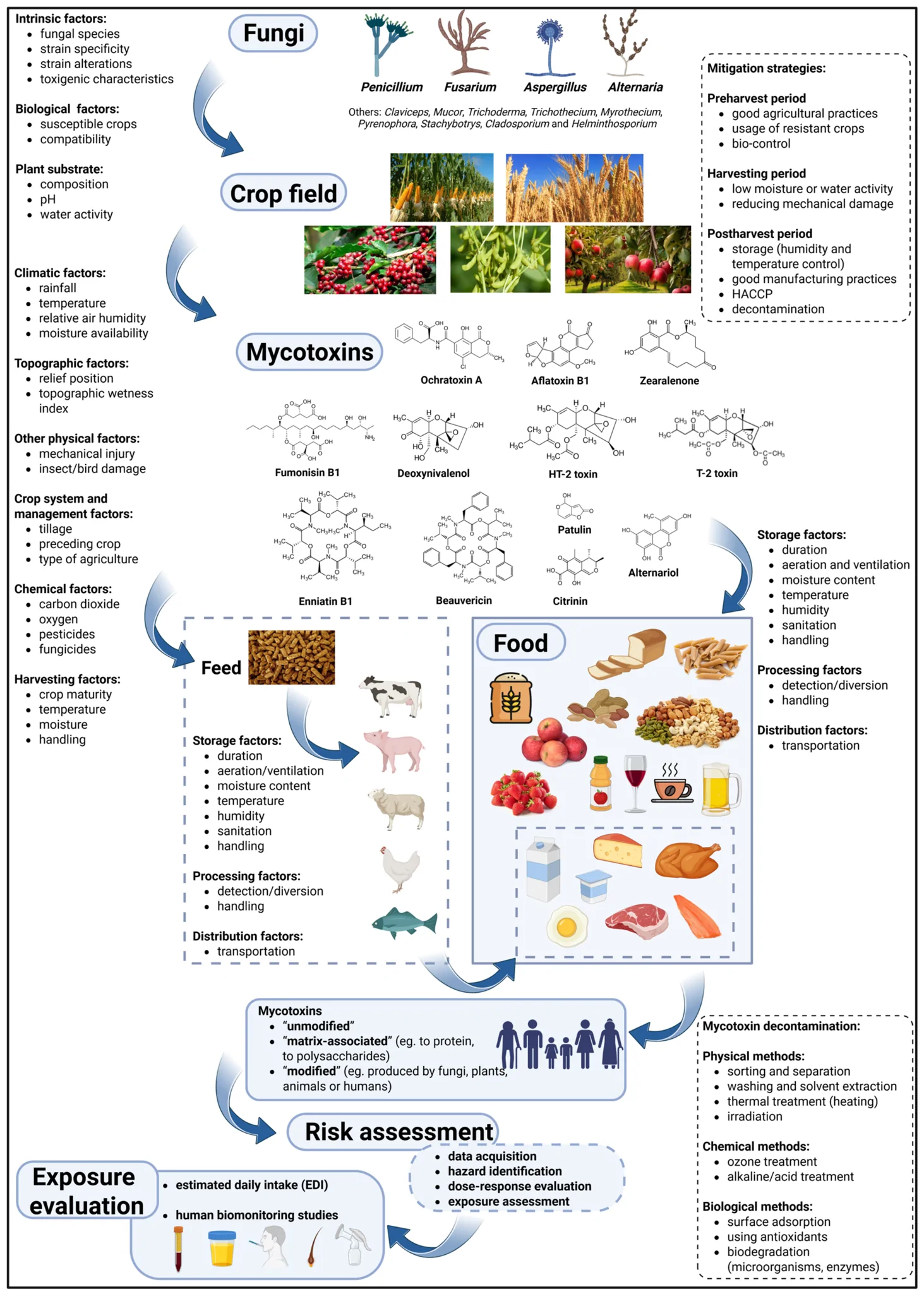
The axis fungi–crop field–mycotoxin (selection)–feed and food–human exposure assessment axis, including the main factors influencing mycotoxin occurrence (left and right), along with some mitigation strategies and detoxification approaches for mycotoxin contamination (right dotted squares). (Created in BioRender. Stanciu, O. M. (2026), accessed on 31 August 2026, https://BioRender.com/gl6sqpv).

Under various stress conditions, mycotoxins can contaminate different plants, and further, they can access the food and feed chain (Figure 1). Their presence represents a problem from several points of view: economic, food safety and food security, public health. According to the - report of the Rapid Alert System for Food and Feed (RASFF), mycotoxins are the third most notified hazard group, with 82% of mycotoxin notifications concerning the detection of AFs, with the most common product category being nuts, nut products and seeds (56%), and the United States of America (USA) being the most reported origin for these notifications (21%). While AFs remain by far the most frequently reported mycotoxins in RASFF notifications, other mycotoxins are also regularly flagged, particularly OTA, followed by DON, PAT, and ZEA, mainly in cereals, dried fruits, nuts, and spices [15]. Regarding food products of animal origin, the main route of contamination is the carry-over of mycotoxins from contaminated feed, particularly into milk and dairy products; contamination of eggs and meat has also been reported, although less frequently [7,8].

Mycotoxins are one of the biggest contributors to food loss, with a direct influence on trade and consequently on the financial level, and an indirect influence on food security [11]. Twenty years ago, the Food and Drug Administration (FDA) estimated the potential financial costs of agricultural damage caused by mycotoxins in the USA at $932 million annually (using a mathematical model) [16], while other recent data proposed a higher value, reaching $1.68 billion per year [17].

The Food and Agriculture Organization (FAO) estimated that approximately 25% of crops around the world are infected with various mycotoxins annually, and around 5 billion people worldwide are exposed to mycotoxins [18,19]. In some cases, this value of 25% has been reported to be even higher, a trend that may reflect both advances in analytical method sensitivity and the growing impact of climate change [20]. Mycotoxins can reach human and animal food chains after direct or indirect contamination [21]. The presence of mycotoxins in food products is considered a global risk, being an important issue in terms of food safety [22]. After traditional [23] or rapid [24] methods of analysis, different mycotoxins were detected in food for direct human consumption, such as cereals (corn [25], rice [26], wheat [27,28], barley [29]), cereal-based products (flour, bread, pasta, biscuits, breakfast cereals) [30], beer [31], lentils, soybeans [32], fruits (apples) [33], nuts (peanuts, tree nuts, almonds, walnuts, pistachios) [34], dried fruits (figs, dates) [35], spices (pepper, paprika, ginger) [36], coffee [37], wine [38], grape juice [39]. Mycotoxins have also been detected as residues in meat and derivatives [18,40], milk and dairy products [41], eggs [42], or seafood [43], as a consequence of their initial presence in feedstuffs [44].

On long-term exposure, according to in vitro and in vivo studies, mycotoxins can induce toxic effects in animals and humans, including endocrine disruptions, oxidative stress, immunosuppression, carcinogenicity, genotoxicity, neurotoxicity, teratogenicity or hepatotoxicity [45,46,47]. Moreover, these toxic effects are highly variable because simultaneous exposure to more than one mycotoxin is frequently reported [4,8], with synergistic, additive, or antagonistic interactions sometimes occurring between mycotoxins. As a result, current research increasingly focuses on predicting the occurrence or toxic activity of mycotoxin combinations using machine-learning models [48,49,50].

Due to their possible negative effects, mycotoxins are regulated in more than 100 countries around the world [8,51], based on the scientific opinions of many international authorities and institutions, including FAO, the World Health Organization (WHO), the FDA, the FAO/WHO Joint Expert Committee on Food Additives of the United Nations (JECFA), the Codex Alimentarius Commission (CAC), and the European Food Safety Authority (EFSA) [8,52]. Also, mycotoxin management is included in the “Farm to Fork strategy” [53] and the European Union Green Deal [54].

Several factors influence the development of mycotoxins in crops during the preharvest period, such as plant substrate, intrinsic factors, environmental and topographic factors, biological factors, crop system, or other physical factors [40,55,56,57]. It is worth mentioning that environmental conditions are becoming some of the most important (with already visible consequences regarding AF contamination), particularly in the context of climate changes recorded (e.g., global warming, extreme phenomena) and predicted for the next 50–100 years [58,59,60,61]. Harvest conditions or significant steps during the post-harvest period (handling, storage, processing, transportation, and distribution) are other key moments related to mycotoxin occurrence in feed and food (Figure 1) [40,62]. Meanwhile, mycotoxin mitigation approaches, including preventive and control strategies, are crucial for food safety, food security, and a sustainable food supply [23,40]. Since many factors during the growing season are unpredictable, postharvest detoxification approaches (physical, chemical, or biological, sometimes combined with generative artificial intelligence) can be used as a last option to deal with mycotoxin problems in food and feed (Figure 1) [23,63,64].

## 2. Mycotoxin Biomonitoring

To minimize exposure risks, most studies focus on the evaluation of mycotoxins in raw materials or products intended for human consumption. The scientific literature comprising numerous studies concerning the occurrence and co-occurrence of mycotoxins in a wide variety of food products, with DON, AFs, OTA and ochratoxin B (OTB) being the most common mycotoxins due to their presence in a wide range of foodstuffs that are consumed worldwide [52]. This approach, which periodically evaluates foods for mycotoxin content as a strategy to reduce mycotoxin exposure through food ingestion, is also based on the fact that some mycotoxins have been legislated by various international or governmental institutions. For example, the FDA has set action levels for total AFs [65] and PAT [66], advisory levels for DON [67], and guidance levels for FUMOs [68], while the EU first introduced maximum permitted levels (MPLs) for OTA, AFs, DON, ZEA, FUMOs, PAT, and CIT in different foodstuffs under Regulation (EC) No 1881/2006 [69], which was later repealed and replaced by Regulation (EU) 2023/915 [70]. More recently, Regulation (EU) 2024/1038 established binding maximum levels (MLs) for the sum of T-2 and HT-2 toxins [71], which were previously subject only to non-binding guidance values. Even though mycotoxin levels in foods most frequently fall within the required MLs, toxicological studies indicate that exposure to these compounds occurs over long periods, is largely involuntary, and is cumulative, as the total intake reflects the sum of the amounts present in each food consumed.

Most human mycotoxin exposure happens through the digestive system when contaminated food is consumed. However, some people can inhale mycotoxins, which mostly happens in workplaces (e.g., workers in the food and feed industries, silos, warehouses, drivers of vehicles designated to transport grain, operators engaged in waste treatment and disposal activities, and maintenance of agricultural, forestry, and animal husbandry machinery) or in water-damaged buildings where mycotoxigenic fungi grow on materials and products [72,73].

**Figure 2:**
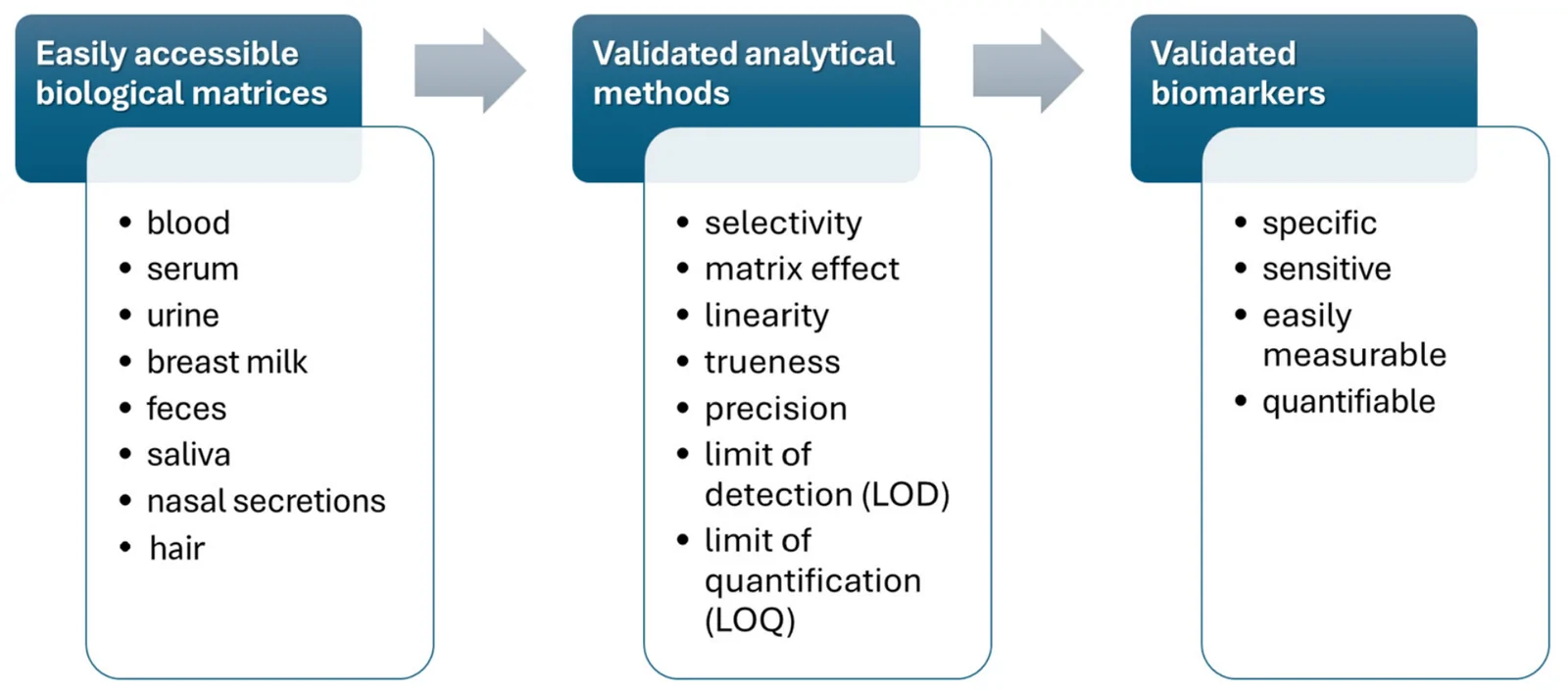
The elements of the human mycotoxin biomonitoring process.

Considering all routes of exposure (inhalation, digestion and dermal), to deliver more accurate mycotoxin exposure data, methods that assess mycotoxins using specific and suitable biomarkers of exposure for the human body (human biomonitoring, HBM) are increasingly applied [52]. To achieve HBM, three elements are necessary (Figure 2): easily accessible biological matrices, validated analytical methods, and validated biomarkers [74].

Mycotoxin biomarkers can be detected: (i) directly (specific biomarkers and validated analytical methods required); (ii) indirectly (quantification of so-called biomarkers of effect, indicating structural or functional changes induced in the body because of toxin exposure); and (iii) non-target (identification of unknown mycotoxin derivatives) [52,75,76]. The process of human mycotoxin biomonitoring includes the detection of the parent compound and/or its main phase I and phase II metabolites (such as glucuronide conjugates) in accessible body fluids or body specimens (Figure 2) [74,77,78,79,80].

Selecting the most appropriate matrix for assessing mycotoxin exposure using HBM is a key step in obtaining reliable results. While matrices such as blood, serum, feces, and hair are difficult to process, purify, and analyze due to the complexity of their composition, with some of them being also invasive (serum or blood), other matrices such as urine samples are easier to collect and analyze, although this matrix also has some limitations [52,77,79]. Urine sampling involves non-invasive procedures, but it must be considered that this type of sample can present daily variations, with optimal sampling requiring a 24-h collection, which is often not feasible in real-world sample collection. Thus, a single sample is most often used in studies. Because each person’s urine volume is unique, the concentration of excreted compounds and metabolites in urine varies. By using a popular method involving normalization with creatinine levels, mycotoxin concentrations in urine can be adjusted, and differences due to urinary volume are minimized. Even so, as creatinine can be influenced by a variety of parameters (such as sex, age, diet, muscle mass, and season), it is still unknown if the mycotoxin/creatinine ratio is the best choice to compare mycotoxin exposure between individuals [81,82].

According to Al-Jaal et al. [83], when HBM is applied to assess mycotoxin exposure, all forms of mycotoxins should be considered, including free mycotoxins and their metabolites and adducts. In fact, mycotoxins can exist in three potential forms: (i) unmodified—known as the parent compound or free form of the mycotoxin, meaning the form biosynthesized by fungal metabolism; (ii) matrix-associated—when mycotoxin is bound to matrix components, resulting in non-covalent complexes (like FUMOs-proteins or OTA-polysaccharides); and (iii) conjugated or masked mycotoxins—as a result of chemical or biological changes in the structure of the parent compound, these modifications being produced depending on various factors by fungi (e.g., ZEA-14-sulfate), bacteria, plants (e.g., DON 3-glucoside), animals and humans (e.g., deepoxy-DON), or during food processing [84,85].

In this study, the analytical methods for determining the occurrence of unmodified or modified mycotoxins in human urine and the results obtained over the past 25 years were presented. Only relevant data was selected, and the studies were classified by mycotoxin classes. At the end of the article, a summary of relevant information about human risk assessment based on mycotoxin biomonitoring in human urine was introduced.

## 3. Analytical Methods for Mycotoxin Analysis in Human Urine

**Figure 3:**
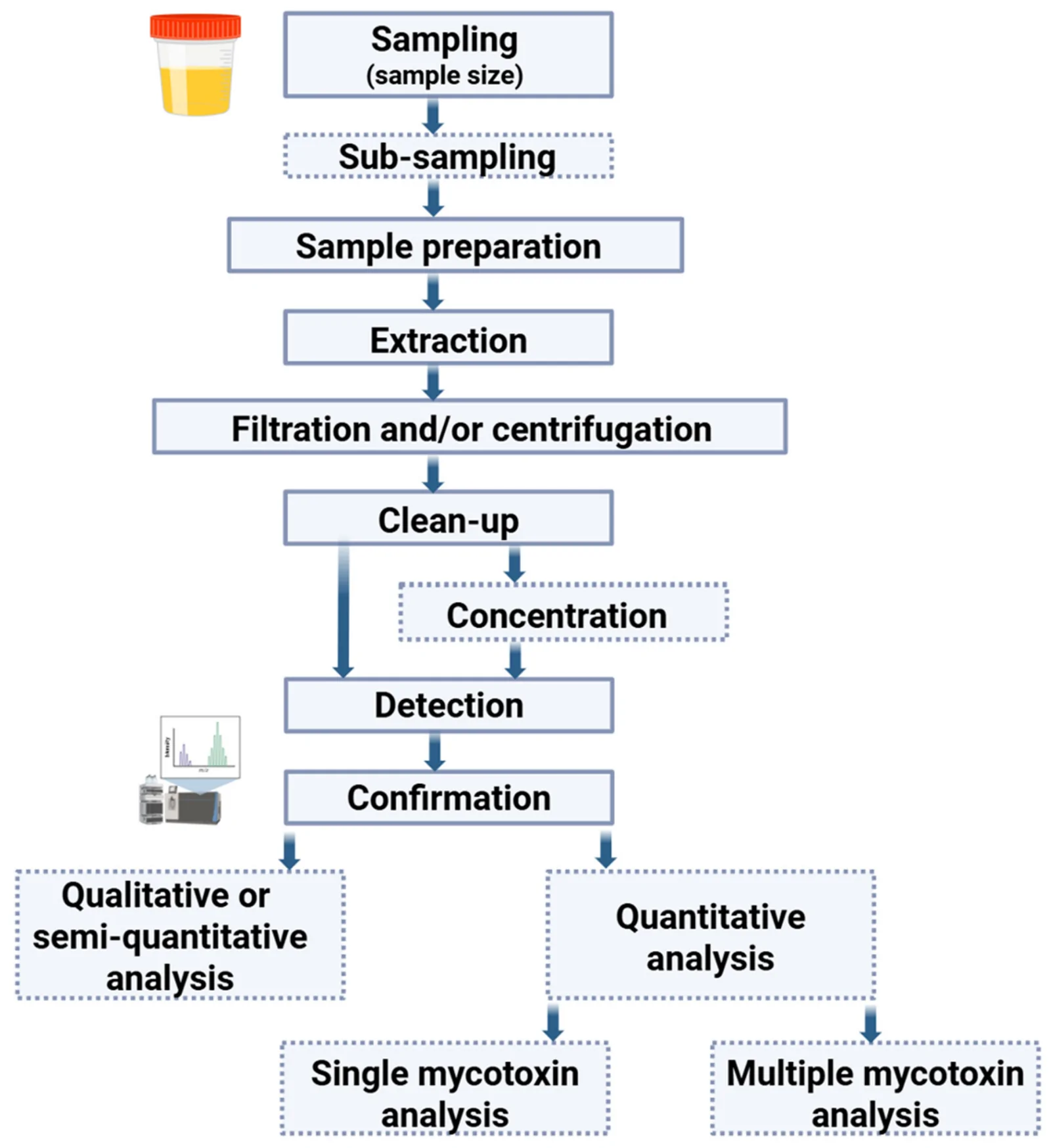
Flow diagram of usual steps involved in mycotoxin analysis in human urine. (Created in BioRender. Stanciu, O. M. (2026), accessed on 8 August 2026, https://BioRender.com/fwlifur).

Mycotoxins excreted in human urine can be analyzed by a wide variety of analytical methods, differentiated by efficiency, precision and sensitivity. The most important steps in processing and analyzing human urine samples to quantify mycotoxins are presented in Figure 3. Each method may have small modifications based on the tools and supplies available. The sample size is quite variable in such studies, considering the willingness of the subjects to participate in this type of study, but also depending on the size of the group from which the selection is made. The precise volume of urine collected is not normally mentioned in the literature, but when urine samples are analytically processed, the sample volume can range between 0.1 mL and 20 mL, depending on the technique and equipment used [86,87]. As preliminary steps, urine samples are centrifuged to eliminate solid particles, and subsequently, an aliquot of the previously centrifuged urine is hydrolyzed, most frequently with β-glucuronidase. When the extraction is performed, various trends can be observed depending on the number of mycotoxins that are targeted for extraction.

Table 1 shows a summary of single and multi-mycotoxin studies in human urine from the last 25 years, including the year of publication, the number of mycotoxins included, a list of mycotoxins, extraction technique, detection method, limits of detection (LODs) and limits of quantification (LOQs), the last two being presented as a range of values, the lowest and the highest levels established during analytical method validation. Most studies conducted between 2001 and 2010 included only one mycotoxin, most often OTA. On the other hand, due to the significant progress in equipment performance, a large number of mycotoxins were included in recent studies, confirming that researchers prefer the simultaneous analysis of numerous compounds in a single run. Recently, mycotoxins and pesticides [88,89] have been included in the same analytical study, or mycotoxins were extracted and analyzed along with dietary phytoestrogens excreted in human urine [90].

During human urine analysis, when one mycotoxin is studied, the extraction methods used are mostly simplistic, a few of them including a clean-up step. For example, when a single mycotoxin analysis is performed, methods such as immunoaffinity (immunoaffinity columns, IACs), liquid–liquid extraction (LLE), solid-phase extraction (SPE), and solid-phase microextraction (SPME) are used. When two or more mycotoxins are evaluated simultaneously in human urine, there are many approaches in the analytical process. For the extraction procedures, IACs and LLE techniques are feasible, but the best analytical findings were achieved using QuEChERS-based procedures, known to be quick, easy, cheap, effective, rugged, and safe, and successfully applied to extract 37 mycotoxins and metabolites from human urine [91]. For mycotoxin quantification in human urine, liquid chromatography (LC) coupled with various detectors (MS; fluorescence detection—FLD; high-resolution mass spectrometry—HRMS; tandem mass spectrometry—MS/MS; multi-wall carbon nanotubes detection—MWCNTs) is the most used, while gas chromatography (GC) [92,93] is very rarely applied (Table 1).

Over the 2001–2010 period, mycotoxin biomonitoring methods evolved from predominantly single-analyte approaches toward more sensitive multi-mycotoxin strategies. This was accompanied by the increasing use of mass spectrometry (MS)-based techniques and lower detection limits, although substantial methodological constraints remained, limiting direct comparison of analytical performance across studies.

Between 2011 and 2020, the number of mycotoxins simultaneously assessed in human urine increased substantially, from panels of 2–11 analytes in the early part of the decade to multi-biomarker methods covering up to 37 mycotoxins and their metabolites by 2019 [91]. This period was marked by a progressive shift from single-analyte approaches, primarily targeting OTA or DON, toward comprehensive multi-mycotoxin panels incorporating major regulated mycotoxins (AFs, OTA, DON, ZEA, FUMOs) alongside their phase I and phase II metabolites, particularly glucuronide conjugates (e.g., DON-3-GlcA, DON-15-GlcA, ZEA-14-GlcA). IAC clean-up and LLE remained the predominant extraction techniques throughout most of the decade, while LC-MS/MS progressively replaced HPLC-FLD as the reference detection method, offering improved sensitivity and the ability to detect multiple analytes simultaneously. From around 2013 onward, simplified “dilute-and-shoot” extraction protocols began to emerge as faster, lower-solvent alternatives to conventional clean-up procedures, reflecting an early trend toward more streamlined sample preparation.

The period between 2021 and 2025 was characterized by further diversification and refinement of analytical strategies, with several studies reporting extended multi-mycotoxin panels comprising over 20 to 36 analytes, including a broader range of emerging mycotoxins (e.g., ENs, BEA, *Alternaria* toxins) alongside classical regulated mycotoxins. LC-MS/MS, including high-resolution variants (LC-HRMS) and quadrupole time-of-flight configurations (LC-ESI-QTOF-MS), consolidated its position as the dominant detection technique, while novel extraction approaches gained traction, including salting-out assisted liquid-liquid extraction (SALLE) [88], online SPE [94], and dried urine spot sampling [95], the latter offering a minimally invasive and field-friendly alternative to conventional urine collection. This period also saw notable expansion of analytical sensitivity, with LOD/LOQ values spanning several orders of magnitude depending on the specific mycotoxin panel and instrumentation used.

Even though several studies have reported the “dilute-and-shoot” technique as a sample preparation method, only one study from 2025 presented the entire process of analysis as an eco-friendly analytical method for mycotoxin analysis in human urine using a straightforward dilute-and-shoot sample preparation, combined with an ultra-high-performance liquid chromatography (UHPLC) coupled to a tandem MS system. Briefly, urine samples obtained after centrifugation (at 4000 rpm for 10 min at 20 °C) were mixed with an internal standard working solution and a mixture of water: acetonitrile (70:30 *v*/*v*) and directly injected into the UHPLC-MS/MS, achieving satisfactory analytical performance and being successfully applied for the analysis of twelve mycotoxins in real human urine samples from Valencian residents (Spain) [96].

LOD ranged between 0.000075 ng/mL [97] and 12 ng/mL [98], while LOQ ranged from 0.00025 ng/mL [97] to 40 ng/mL (Table 1) [98].

**Table 1:** Summary of analytical parameters of validated methods for the analysis of free mycotoxins, their metabolites or adducts in human urine (ordered by year of publication, from 2001 to 2025).

Mycotoxins (Number: Name)	Extraction Details	Detection Method	LOD (Range) ng/mL	LOQ (Range) ng/mL	Year of Publication	Ref.
**10:** OTA, 4-OH-OTA, OTB, AFB1, AFM1, AFL, AFB2, AFM2, AFG1, AFG2	LLE/SPE	HPLC-FLD	NA	NA	2001	[99]
**1:** OTA	IAC	HPLC	0.005	NA	2001	[100]
**1:** OTA	IAC	HPLC-FLD	NA	0.01	2001	[101]
**1:** DON	IAC	HPLC-ESI-MS	NA	4	2003	[102]
**1:** OTA	LLE/SPE	HPLC	0.3	0.9	2003	[103]
**1:** OTA	IAC	HPLC	0.004	0.006	2005	[104]
**1:** OTA	IAC	HPLC-FLD	NA	0.02	2006	[105]
**1:** OTA	SPME	LC-FLD	0.01	0.05	2007	[106]
**1:** DON	IAC	HPLC-MS	NA	0.6	2007	[107]
**1:** OTA	SPME	LC-MS/MS	0.3	0.7	2008	[108]
**1:** OTA	IAC	HPLC-FLD	NA	0.007	2008	[109]
**6:** ZEA, α-ZEL, β-ZEL, α-ZAL, β-ZAL, ZAN	SPE	HPLC-MWCNTs	1.3–1.4	4.2–4.8	2008	[110]
**1:** FB1	SPE	HPLC-ESI-MS	0.02	NA	2008	[111]
**1:** OTA	IAC	HPLC-FLD	NA	0.008	2009	[112]
**2:** OTA, OTα	LLE	LC-FLD	0.02	0.05	2010	[113]
**1:** OTA	IAC	HPLC-FLD	NA	0.008	2010	[114]
**2:** FB1, FB2	IAC	LC-MS/MS	5	10	2010	[115]
**5:** OTA, OTα, AFM1, FB1, FB2	IAC	LC-MS/MS	0.001–0.045	0.004–0.135	2010	[116]
**1:** OTA	IAC	HPLC-FLD	0.006	0.018	2010	[117]
**1:** DON	IAC	HPLC-MS	0.5	NA	2010	[118]
**2:** OTA, OTα	LLE	HPLC	0.023–0.034	0.076–0.112	2011	[119]
**11:** DON, T-2, HT-2, ZEA, OTA, AFB1, AFB2, AFG1, AFG2, FB1, FB2	IAC	LC-MS/MS	0.4–10	1.2–35	2011	[120]
**1:** DON-3-GlcA	LLE (dilute and shoot)	LC-MS/MS	3	10	2011	[121]
**7:** AFM1, OTA, DON, DOM-1, α-ZEL, β-ZEL, FB1	IAC-SPE	LC-MS/MS	0.01–2.2	0.02–4.4	2011	[122]
**1:** OTA	IAC	HPLC-FLD	0.0024	0.008	2012	[123]
**15:** DON, DON-3-GlcA, DON-15-GlcA, DOM-1, NIV, T-2, HT-2, ZEA, ZEA-14-GlcA, α-ZEL, β-ZEL, FB1, FB2, OTA, AFM1	SPE	LC-ESI-MS/MS	0.05–10	0.17–67	2012	[124]
**18:** AFB1, AFM1, AFB1-N7-Gua, OTA, OTα, 4-OH-OTA, DON, DOM-1, DON-3-GlcA, ZEA, α-ZEL, β-ZEL, ZEA-14-GlcA, T-2, HT-2, CIT, FB1, HFB1	LLE-SPE	UHPLC-MS/MS	0.01–3.65	0.02–7.3	2012	[125]
**2:** CIT, DH-CIT	IAC-SPE	LC–MS/MS	0.02–0.05	0.05–0.1	2013	[126]
**15:** FB1, FB2, AFM1, OTA, DON, DON-3-GlcA, DON-15-GlcA, DOM-1, NIV, T-2, HT-2, ZEA, ZEA-14-GlcA, α-ZEL, β-ZEL	LLE (dilute and shoot)	LC–MS/MS	0.002–0.45	0.007–1.51	2013	[127]
**14:** AFM1, FB1, FB2, OTA, DON, DON-3-GlcA, DOM-1, NIV, T-2, HT-2, ZEA, ZEA-14-GlcA, α-ZEL, β-ZEL	LLE (dilute and shoot)	LC–MS/MS	0.05–12	0.15–40	2014	[98]
**15:** DOM-1, DON, 3-AcDON, FUS-X, DAS, NIV, NEO, HT-2, T-2, ZAN, α-ZAL, β-ZAL, ZEA, α-ZEL, β-ZEL	d-SPE	GC-QqQ-MS/MS	0.12–4	0.25–8	2014	[93]
**23**: DON, DON-3-GlcA, T-2, HT-2, HT-2-4-GlcA, FB1, AFB1, AFB2, AFG1, AFG2, AFM1, ZEA, ZAN, α-ZEL, β-ZEL, ZEA-14-GlcA, ZAN-14-GlcA, α-ZEL-14-GlcA, β-ZEL-14-GlcA, OTA, OTα, ENB, DH-CIT	dilute and shoot	LC-MS/MS	0.0005–0.3125	0.0013–0.3125	2014	[128]
**2:** CIT, DH-CIT	IAC	LC–MS/MS	0.02–0.05	0.05–0.1	2015	[129]
**23:** DON, DON-3-GlcA, T-2, HT-2, HT-2-4-GlcA, FB1, AFB1, AFB2, AFG1, AFG2, AFM1, ZEA, ZAN, α-ZEL, β-ZEL, ZEA-14-GlcA, ZAN-14-GlcA, α-ZEL-14-GlcA, β-ZEL-14-GlcA, OTA, OTα, ENB, DH-CIT	dilute and shoot	LC-MS/MS	0.000125–0.45	0.0005–0.9	2015	[130]
**32**: AFB1, AFB2, AFG1, AFG2, OTA, AFM1, DAS, Fus-X, 3-AcDON, 15-AcDON, β-ZEL, α-ZEL, CIT, DH-CIT, OTα, DOM-1, FB1, FB2, FB3, DON, ZEA, T-2, HT-2, DON-3-GlcA, DOM-GlcA, ZEA-14-GlcA, β-ZEL-7-GlcA, β-ZEL-14-GlcA, α-ZEL-7-GlcA, α-ZEL-14-GlcA, 15-AcDON-3-GlcA, 3-AcDON-15-GlcA	Filter-shot/ IAC (only for OTA, CIT, AFM1)	LC-MS/MS	0.001–0.2	0.003–0.5	2015	[131]
**9:** CIT, DH-CIT, DON, DOM-1, OTA, OTα, ZEA, α-ZEL, β-ZEL	IAC/LLE (only for OTA)	HPLC/LC–MS/MS	0.002–0.150	0.005–0.300	2016	[132]
**2:** AFM1, AFB1-N7-Gua	IAC	HPLC-FLD	0.000075–0.003	0.00025–0.01	2016	[97]
**2:** ZEA, α-ZEL	SLE	UPLC-MS/MS	NA	NA	2016	[90]
**3:** OTA, OTA-8-GlcA, open lactone OTA-8-GlcA	LLE	LC–MS/MS	0.03–0.5	0.05–1	2017	[133]
**2:** DON, DOM-1	IAC	LC-MS/MS	0.12–0.25	NA	2017	[134]
**2:** OTA, OTα	LLE	HPLC-FLD	0.01	0.02	2017	[135]
**12:** NIV, DON, DOM-1, OTA, AFM1, CIT, DH-CIT, FB1, ZEA, α-ZEL, β-ZEL, AOH	IAC-SPE	UHPLC-MS/MS	0.0003–0.3	0.001–0.5	2018	[136]
**6:** ZEA, ZAN, α-ZEL, β-ZEL, α-ZAL, β-ZAL	LLE-SPE	UHPLC-MS/MS	0.02–0.06	0.05–0.2	2018	[137]
**3:** DON, DON-GlcA, DOM-1	IAC	LC-QTOF-MS	NA	0.25–0.5	2018	[138]
**26:** AFB1, AFB2, AFG1, AFG2, AFB1-lysine, AFM1, AFM2, OTA, OTα, FB1, T-2, HT-2, DON, 3-AcDON, 15-AcDON, DON-3-GlcA, DON-15-GlcA, FUS-X, ZEA, ZAN, α-ZEL, β-ZEL, α-ZAL, β-ZAL, ZEA-14-GlcA, ZAN-14-GlcA	LLE	UHPLC-MS/MS	0.03–0.5	0.1–1	2019	[139]
**3:** OTA, CIT, DH-CIT	IAC	HPLC-FLD	0.001–0.05	0.002–0.1	2019	[140]
**37:** NIV, DON, DOM-1, 3-AcDON, 15-AcDON, DON-3G, CIT, OTA, OTα, T-2, HT-2, T-2 triol, T-2 tetraol, FB1, FB2, FB3, HFB1, ZEA, ZAN, α-ZEL, β-ZEL, α-ZAL, β-ZAL, roquefortine C, DAS, NEO, FUS-X, STG, PAT, AOH, AME, DON-3-GlcA, DON-15-GlcA, α-ZEL-GlcA, β-ZEL-GlcA, ZEA-14-sulfate, ZEA-14-GlcA	QuEChERS-based procedure	LC–MS/MS	0.01–2.6	0.02–4.9	2019	[91]
**2:** DON, DOM-1	IAC	LC-MS	0.25	0.5	2019	[141]
**3:** AFM1, DON, DOM-1	IAC	HPLC-FLD (AFM1) LC–MS/MS (DON, DOM-1)	0.0017–0.16	0.005–0.3	2020	[142]
**6:** ZEA, ZAN, α-ZEL, β-ZEL, α-ZAL, β-ZAL	IAC	LC–MS/MS	0.02–0.06	NA	2020	[143]
**2:** PAT, CIT	QuEChERS-based procedure	LC–MS/MS	0.14–1.67	0.2–2.65	2020	[144]
**2:** DON, AFM1	IAC	LC–MS/MS	0.002–0.25	0.005–0.5	2020	[145]
**10:** DON, 3-AcDON, 15-AcDON, FUS-X, NIV, NEO, ZEA, ZAN, T-2, HT-2	DLLME	GC–MS/MS	0.12–4	0.25–8	2021	[146]
**11:** AFB1, AFM1, OTA, OTα, DON, ZEA, α-ZEL, β-ZEL, FB1, HT-2, T-2	IAC, SPE (only for DON)	LC-HRMS	NA	0.01–0.5	2021	[147]
**12:** FB1, FB2, DON, DOM-1, ZEA, α-ZEL, β-ZEL, OTA, AFM1, T-2, HT-2, NIV	IAC + HLB column	LC–MS/MS	0.0003–6.25	0.001–20.8	2021	[148]
**14:** AFB1, AFB2, AFG2, AFM1, CIT, CPA, FB1, OTA, OTB, STG, T-2, α-ZEL, β-ZEL, roquefortine	LLE	HPLC–ESI–HRAMS	NA	0.2–0.5	2021	[149]
**4:** DON, DOM-1, DON-3-GlcA, DON-15-GlcA	IAC	LC-HRMS	0.005–0.009	0.015–0.27	2021	[150]
**1:** OTA	IAC	HPLC-FLD	0.007	0.01	2021	[151]
**9:** AFB1, AFM1, OTα, OTA, DON, DOM-1, ZEA, α-ZEL, β-ZEL	SPE	UHPLC-MS/MS	0.08–6.6	0.1–20.1	2021	[152]
**11:** AFM1, ALT, AME, AOH, CIT, DH-CIT, FB1, OTA, ZEA, α-ZEL, β-ZEL	SPE	UHPLC-MS/MS	0.0036–0.27	0.012–0.91	2021	[153]
**14:** CIT, DH-CIT, DON, FB1, T-2, HT-2, OTA, 2′R-OTA, OTα, TeA + allo-TeA, ZEA, ZAN, α-ZEL, β-ZEL	dried urine spots/ dilute and shoot	HPLC–ESI-MS/MS	0.004–1.4	0.013–4.5	2021	[95]
**6:** AFB2, AFG2, OTA, OTB, ZEA, α-ZEL	QuEChERS-based procedure	LC-ESI-QTOF-MS	1.5–5	3–10	2022	[87]
**1:** DON	IAC	HPLC-MS/MS	NA	0.3	2023	[154]
**1:** DON	NA	HPLC-MS/LC-HRMS	0.04–0.2	0.05–0.5	2024	[155]
**23:** AFB1, AFB2, AFG1, AFG2, AFM1, DON, DOM-1, 15-AcDON, 3-AcDON, ZEA, α-ZEL, β-ZEL, ZAN, OTα, OTA, OTB, BEA, ENA, ENA1, ENB, ENB1, T-2, HT-2	SALLE	LC-ESI-QTOF-MS	NA	0.1–5	2024	[88]
**22**: 15-AcDON, 3-AcDON, AFB1, AFB2, AFG1, AFG2, AFM1, α-ZEL, β-ZEL, DOM-1, DON, ENA, ENA1, ENB, ENB1, HT-2, T-2, OTα, OTA, OTB, ZEA, ZAN	SALLE	LC-ESI-QTOF-MS	0.01–0.750	0.03–2.5	2024	[89]
**1:** AFM1	filtration and dilution	ELISA	0.988	NA	2025	[156]
**10:** AFM1, OTA, FB1, FB2, ZEA, α-ZEL, β-ZEL, DON, T-2, HT-2	IAC	UPLC-MS/MS	0.01–0.46	0.03–1.32	2025	[157]
**3:** T-2, HT-2, T-2 triol	IAC	UPLC-MS/MS	0.013–0.10	0.05–0.30	2025	[158]
**12:** AFB1, AFB2, AFG1, AFG2, AFM1, ZEA, ZAN, OTA, AME, CIT, STG, AOH	dilute and shoot	UHPLC-MS/MS	NA	0.005–0.5	2025	[96]
**36:** AFM1, STG, FB1, FB2, CIT, DH-CIT, OTA, 2’R-OTA, OTα, 10-OH-OTA, OTB-NAC, ZEA, ZEA-14-GlcA, α-ZEL, β-ZEL, α-ZEL-14-GlcA, β-ZEL-14-GlcA, ZAN-14-GlcA, T-2, HT-2, HT-2-3-GlcA, HT-2-4-GlcA, DON, DON-3-GlcA, ALT, AME, AOH, TeA + allo-TeA, CPA, PENA, PAX, BEA, ENA, ENA1, ENB, ENB1	online SPE	LC-MS/MS	0.001–0.833	0.002–5.0	2025	[94]
**10:** AFM1, FB1, OTA, DON, CIT, NIV, ZEA, DHC, α-ZEL, β-ZEL	SPE	UHPLC-ESI-MS/MS	0.0005–0.05	0.01–0.5	2025	[159]
**10:** AME, AOH, ZEA, ZAN, α-ZAL, β-ZAL, α-ZEL, β-ZEL, ZEA-14-GlcA, ZEA-14-sulfate	LLE	UHPLC-MS/MS	0.0029–0.22	0.010–0.74	2025	[160]

DLLME: dispersive liquid–liquid microextraction; d-SPE: dispersive solid-phase extraction; ELISA: enzyme-linked immunosorbent assay; GC–MS/MS: gas chromatography–tandem mass spectrometry; GC–QqQ–MS/MS: gas chromatography–triple quadrupole–tandem mass spectrometry; HLB: hydrophilic–lipophilic balanced; HPLC: high-performance liquid chromatography; HPLC-ESI-MS: high-performance liquid chromatography–electrospray ionization–single quadrupole mass spectrometry; HPLC-FLD: high-performance liquid chromatography: fluorescence detection; HPLC-MWCNTs: high-performance liquid chromatography–multi-wall carbon nanotubes detection; HPLC-MS: high-performance liquid chromatography–mass spectrometry; IAC: immunoaffinity column; LC-ESI-QTOF-MS: liquid chromatography coupled to quadrupole time of flight mass spectrometry with electrospray ionization; LC-FLD: liquid chromatography–fluorescence detection; LC-HRMS: liquid chromatography–high-resolution mass spectrometry; LC-MS/MS: liquid chromatography–tandem mass spectrometry; LLE: liquid–liquid extraction; LOD: limit of detection; LOQ: limit of quantification; MS: mass spectrometry; QuEChERS: Quick, Easy, Cheap, Effective, Rugged, Safe; SALLE: salting-out assisted liquid–liquid extraction; SLE: supported liquid extraction; SPE: solid-phase extraction; SPME: solid-phase microextraction; UHPLC-MS/MS: ultra-high performance liquid chromatography–tandem mass spectrometry; NA: not available./AFB1: aflatoxin B1; AFB1-N7-Gua: aflatoxin B1-N7-guanine; AFB2: aflatoxin B2; AFG1: aflatoxin G1; AFG2: aflatoxin G2; AFL: aflatoxicol; AFM1: aflatoxin M1; AFM2: aflatoxin M2; ALT: altenuene; AME: alternariol monomethyl ether; AOH: alternariol; BEA: beauvericin; CIT: citrinin; CPA: cyclopiazonic acid; DH-CIT: dihydrocitrinone; DAS: diacetoxyscirpenol; 3-AcDON: 3-acetyl-deoxynivalenol; 15-AcDON: 15-acetyl-deoxynivalenol; 3-AcDON-15-GlcA: 3- acetyl-deoxynivalenol-15-glucuronide; 15-AcDON-3-GlcA: 15-acetyl-deoxynivalenol-3-glucuronide; DOM-1: deepoxy-deoxynivalenol; DOM-GlcA: deepoxy-deoxynivalenol-glucuronides; DON: deoxynivalenol; DON-3G: deoxynivalenol-3-glucoside; DON-GlcA: deoxynivalenol-glucuronides; DON-3-GlcA: deoxynivalenol-3-O-β-glucuronide; DON-15-GlcA: deoxynivalenol-15-O-β-glucuronide; ENA: enniatin A; ENA1: enniatin A1; ENB: enniatin B; ENB1: enniatin B1; FB1: fumonisin B1; FB2: fumonisin B2; FB3: fumonisin B3; FUS-X: fusarenone-X; HFB1: hydrolyzed fumonisin B1; HT-2: HT-2 toxin; HT-2-3-GlcA: HT-2 toxin-3-glucuronide; HT-2-4-GlcA: HT-2 toxin-4-glucuronide; NEO: neosolaniol; NIV: nivalenol; OTA: ochratoxin A; OTα: ochratoxin α; OTA-8-GlcA: ochratoxin A-8-β-glucuronide; 4-OH-OTA: 4-hydroxy-ochratoxin A; 10-OH-OTA: 10-hydroxy-ochratoxin A; OTB: ochratoxin B; OTB-NAC: ochratoxin-N-acetyl-L-cysteine; PAT: patulin; PAX: paxilline; PENA: penitrem A; 2’R-OTA: 2′R-ochratoxin A; STG: sterigmatocystin; T-2: T-2 toxin; TeA: tenuazonic acid; α-ZAL: α-zearalanol; β-ZAL β-zearalanol; ZAN: zearalanone; ZAN-14-GlcA: zearalanone-14-glucuronide; ZEA: zearalenone; ZEA-14-GlcA: zearalenone-14-O-β-glucuronide; ZEA-14-sulfate: zearalenone-14-sulfate; α-ZEL: α-zearalenol; α-ZEL-GlcA: α-zearalenol-glucuronides; β-ZEL: β-zearalenol; β-ZEL-GlcA: β-zearalenol-glucuronides.

**Figure 4:**
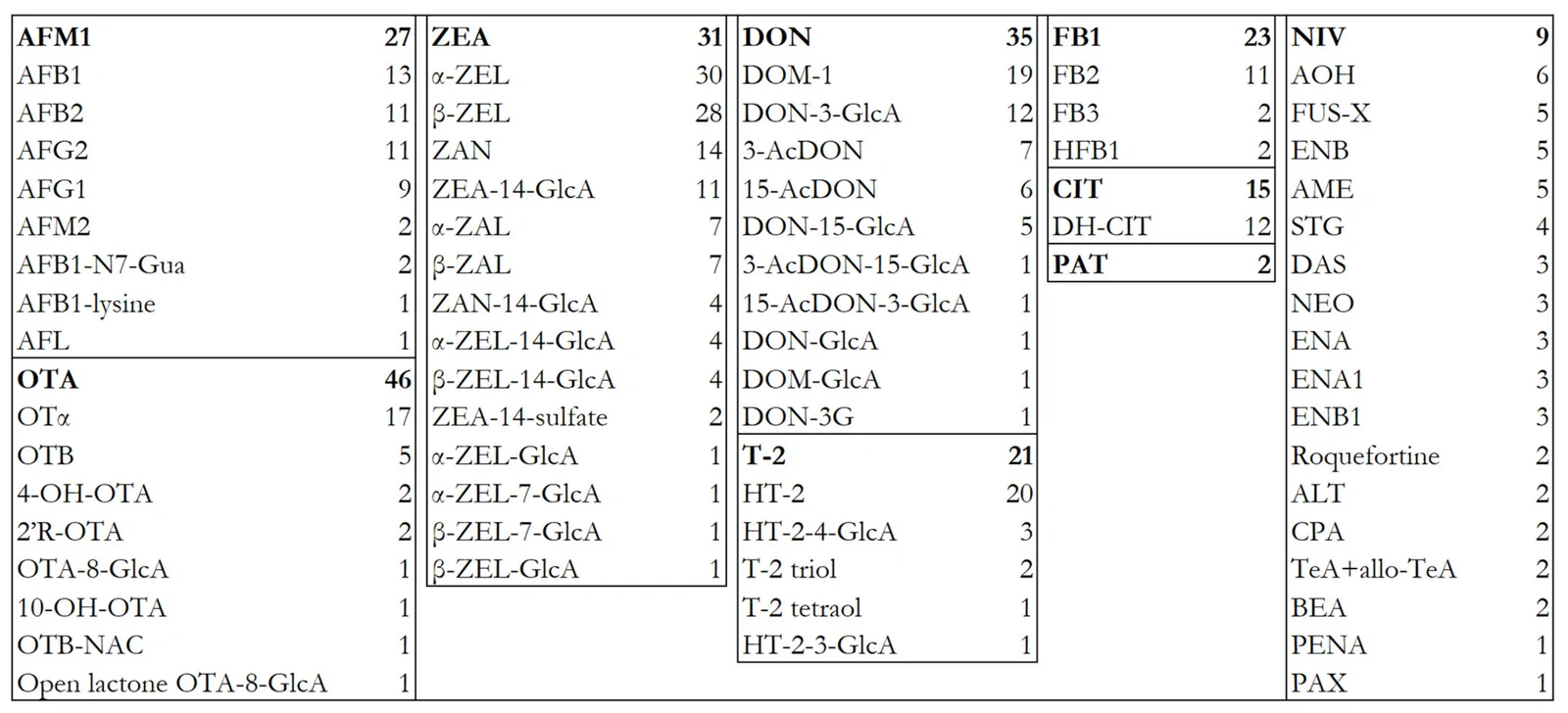
Number of studies analyzing various free mycotoxins, metabolites, or adducts in human urine (from a total of 73 studies published between 2000 and 2025). Detailed data is presented in Table 1. Abbreviations are defined in the footnote of Table 1.

The most analyzed mycotoxins in human urine appear to be OTA, DON, and ZEA, along with their metabolites, while PAT and emerging mycotoxins such as NEO, NIV, DAS, FUS-X, ALT, TeA or ENs are less frequently evaluated (Figure 4). This fact is probably influenced by the lack of legislative regulations and still incomplete toxicological studies for the emerging mycotoxins, the reduced availability of analytical standards, or various analytical challenges that arise during simultaneous multi-compound analysis.

Among the studies for which the country of investigation was explicitly reported, more than half (54%) were conducted in Europe, with Portugal, the United Kingdom, and Germany contributing the largest share, followed by Spain and Italy. Asia accounted for approximately a quarter of the studies (24%), predominantly from China, Bangladesh, and Turkey. Africa was represented mainly by studies from Nigeria, accounting for 10% of the total, while North America (5%) and South America (3%) contributed comparatively fewer studies. An additional 5% of studies were multinational, spanning several European countries or combining European and Asian populations.

## 4. Occurrence of Mycotoxins in Human Urine—Step-by-Step Review

### 4.1. Aflatoxins (AFs)

**Figure 5:**
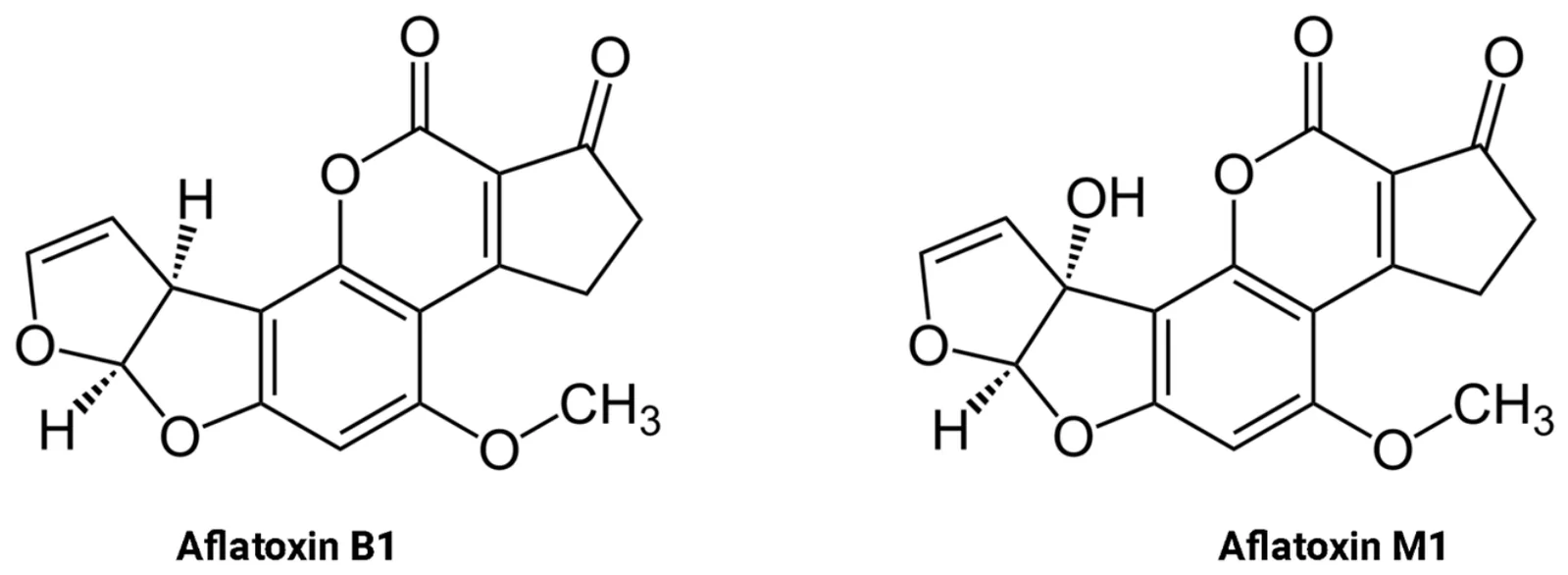
Chemical structures of two of the most studied aflatoxins.

AFs (Figure 5) are some of the best-known mycotoxins. Chemically, AFs are bifuranocoumarin derivatives with a cyclopentenone (AFs from group B) or a δ-lactone ring (AFs from group G), grouped and named based on their properties under ultraviolet irradiation, where AFs from group B (AFB1 and aflatoxin B2, AFB2) emit blue fluorescence (450 nm), and AFs from group G (aflatoxin G1, AFG1, and aflatoxin G2, AFG2) emit green fluorescence (425 nm) [161]. Among the compounds in this class, AFM1 (the hydroxylated metabolite of AFB1) and aflatoxin M2 (AFM2, the hydroxylated metabolite of AFB2) have also been intensively studied, with AFM1 being known as “the milk toxin” due to its predominant presence in milk because of AFB1 metabolism and its excretion into milk in this chemically modified form [161,162].

AFs are produced mostly by *Aspergillus flavus* and *Aspergillus parasiticus*, but also by other *Aspergillus* species (*A. nomius*, *A. arachidicola*, *A. bombycis*, *A. pseudotamarii*, *A. minisclerotigenes*, *A. rambellii*) or by some *Emericella* species (*E. astellata*, *E. venezuelensis*, *E. olivicola*) [23,40]. Generally, tropical, warm, arid and semi-arid regions provide optimum conditions (temperatures between 10 and 43 °C and a water activity (a_w_) between 0.80 and 0.99) for the growth of AF-producing fungi [7,36].

AFs can be detected in a wide variety of plant-based or animal products, including maize, wheat, rice, sorghum, spices (chili peppers, black pepper, coriander, turmeric, ginger), groundnuts, tree nuts, almonds, oilseeds (peanut, sunflower, soybean, cotton), dried fruits, milk and dairy products, eggs, and meat [40]. AFs are known to be hepatocarcinogenic (due to lipid peroxidation and oxidative damage to DNA), mutagenic, teratogenic, and immunosuppressive to most animal species [162], thus the International Agency for Research on Cancer (IARC) has classified AFs, including AFB1, AFB2, AFG1, AFG2 and AFM1, as carcinogenic to humans (Group 1), with AFB1 being considered the most potent [163,164].

During its metabolization, AFB1, the most frequent food contaminant among the AFs, is transformed into AFB1-8,9-epoxide (its reactive form), and afterwards can bind to cellular macromolecules, leading to the appearance of a DNA adduct, AFB1-N7-guanine (AFB1-N7-Gua), which is also detected in human urine [72]. Various researchers indicate that both AFB1-N7-Gua and AFM1 in urine are strongly correlated with AF intake [165]. Thus, both compounds act as good biomarkers of relatively recent AF exposure.

AFs are the fourth most frequently included mycotoxins in studies on human urine samples, after OTA, DON, and ZEA (Figure 4). AFM1 appears to be the most studied mycotoxin from AFs, with the highest concentration detected being 374 ng/mL in a study from Sierra Leone (Table 2) [99]. Interestingly, in the same study, aflatoxicol (AFL) was the most frequently detected (62%) in both sexes, with a slightly higher incidence in men. AFM2 was detected in 51% of boys and 44% of girls, whereas AFB1 was present in 49% and 35% of girls and boys, respectively [99]. Higher male exposure to AFs was also observed in a recent study from the United Arab Emirates (UAE), with males demonstrating a higher mean and maximum AFM1 concentration (mean 0.912 ng/mg; range: 0.0–15.40 ng/mg) compared to females (mean 0.676 ng/mg; range: 0.0–6.42 ng/mg), although statistical testing found no significant gender-based difference in aflatoxin concentration [156].

### 4.2. Ochratoxin A (OTA)

**Figure 6:**
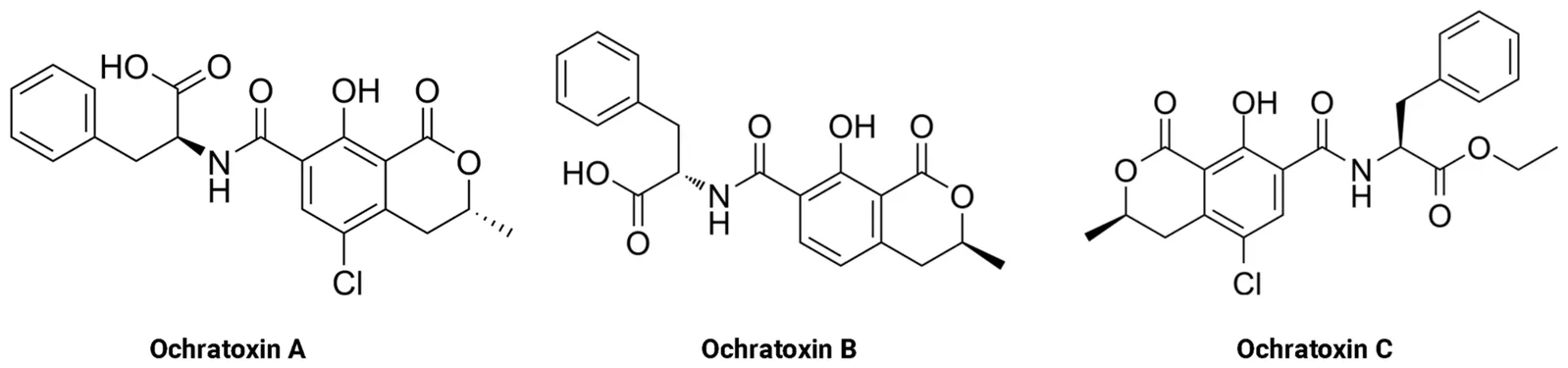
Chemical structures of ochratoxins.

Ochratoxins can be produced by various species of *Aspergillus* (mostly *A. ochraceus*, but also *A. niger* and *A. carbonarius*) and *Penicillium* (*P. verrucosum*, together with *P. nordicum* and *P. viridicatum*) [23]. The most potent toxin among the ochratoxins is OTA (its chemical name being L-phenylalanine-N-[(5-chloro-3,4-dihydro-8-hydroxy-3-methyl-1-oxo-1*H*-2-benzopyrane-7-yl)carbonyl] -(R)-isocoumarin), but other compounds have also been studied over time, including OTB, ochratoxin C (OTC) (Figure 6), the isocoumaric derivative of OTA, ochratoxin α (OTα), and its dechloro analog, ochratoxin β (OTβ) [166]. Generally, *A. ochraceus* can produce OTA mostly in hot-tropical regions (with an optimal temperature of 31 °C and an a_w_ of around 0.80), whereas *P. verrucosum* prefers cool-temperate conditions to grow (with an optimal temperature of 20 °C and an a_w_ of around 0.86) [8,36,167].

Ochratoxins have been detected in cereals (particularly in wheat, barley, oats, and rye), dried vine fruit, wine, beer, coffee, chocolate, spices, raisins, grape juice, and also in pork, poultry, and dairy products [40,168]. Throughout the last half-century, a lot of experimental studies have been carried out to show the negative effects of OTA and to demonstrate the link between OTA exposure and various diseases. Thus, it has been found that OTA can be hepatotoxic, nephrotoxic, neurotoxic, teratogenic, immunotoxic, and sometimes genotoxic in several species of animals. However, toxicity results may be influenced by the sex and type of the animal used in the animal tests or the type of cells used during in vitro studies [166]. Even so, there is a clear consensus regarding the possible toxicity of OTA, so it has been classified by the IARC as a Group 2B possible human carcinogen, based on demonstrated carcinogenicity in animal studies [164,169].

OTA is metabolized by various CYP enzymes, with detected metabolites of OTA including OTα, 4-hydroxy-OTA (4-OH-OTA) and 10-hydroxy-OTA (10-OH-OTA), which are formed by OTA peptide bond hydrolysis [72].

According to the literature studied, the most analyzed mycotoxins in human urine appear to be OTA and its metabolites, with 63% of the studies investigating mycotoxins in human urine published between 2000 and 2025 including OTA in their evaluation (Figure 4). This is likely related to the interest generated by its demonstrated toxicity, as well as its frequency of occurrence in food and feed. In urine samples, the most analyzed forms for assessing the presence of OTA are OTA and OTα (Table 3). Generally, concentrations of metabolites from the OTA metabolism cascade are found in urine at values below 1 ng/mL.

When low LOQs are reached (between 0.001 and 0.02 ng/mL), OTA can be detected in up to 100% of the analyzed samples, as can be observed in studies conducted on subjects from Germany [113,132,135], Korea [116], France [147], Hungary [148], and Lebanon [151]. Also, using a new online SPE-LC-MS/MS method applied to 50 pregnant women in Bangladesh, Kuhn et al. (2025) [94] detected OTA in 98% of samples. The highest levels of OTA in human urine (59–148 ng/mL) were found in samples from boys and girls in Sierra Leone [99], with a high maximum level of OTB (218 ng/mL) detected in the same study (Table 3), while the highest concentration of OTα (15 ng/mL) was registered in a study from Belgium [125].

### 4.3. Zearalenone (ZEA)

**Figure 7:**
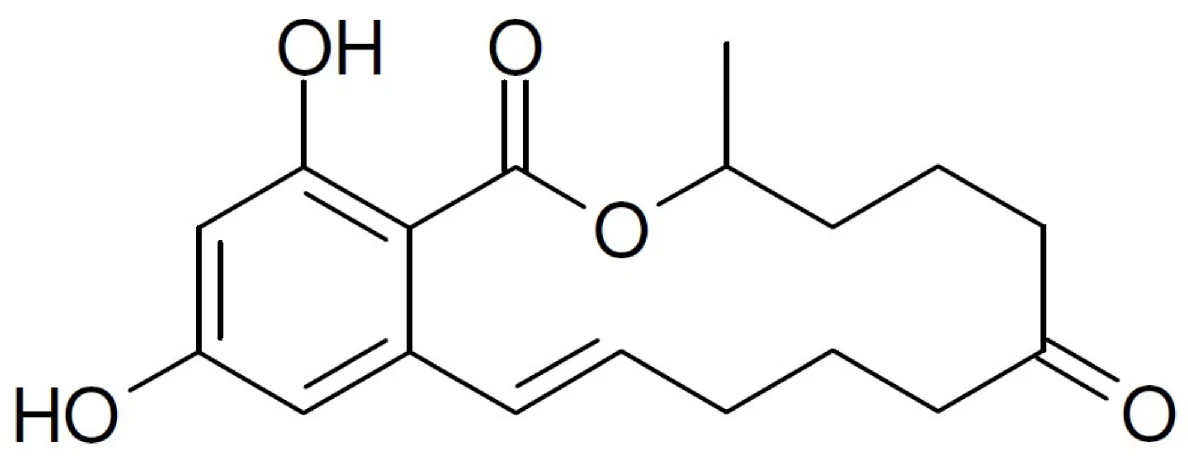
Chemical structure of zearalenone.

ZEA, chemically known as 6-[10-hydroxy-6-oxo-trans-1-undecenyl]-beta-resorcylic acid lactone (Figure 7), is a non-steroidal estrogenic mycotoxin produced by various *Fusarium* species (*F. graminearum*, *F. culmorum*, *F. crookwellense*, *F. equiseti*, *F. sporotrichioides*, *F. cerealis*, *F. incarnatum*) [40]. ZEA requires an optimal temperature of 25 °C and an a_w_ of around 0.96 [36], and it naturally occurs in barley, oats, wheat, rice, sorghum, cereal-based products, sesame, and soybeans [23,40].

ZEA is important because animals and humans are chronically exposed to it, as it is common and frequently found in combination with other mycotoxins. ZEA and its chemical derivatives are the only known mycotoxins with primarily estrogenic effects (being classified as endocrine-disrupting chemicals), with the literature referring to them as mycoestrogens or xenoestrogens [171]. Thus, ZEA and its metabolites can induce various alterations, such as alterations in the reproductive tract, uterus enlargement, low fertility, increased embryofetal resorptions, increased litter size, and modifications in progesterone and estradiol serum concentrations. Other toxic effects mentioned in the literature for ZEA or its metabolites, due to the lipid peroxidation enhancement, include hepatotoxicity, immunotoxicity, and carcinogenicity to various mammalian species [172,173]. Even so, the IARC has classified ZEA as Group 3, “not classifiable as to its carcinogenicity to humans” [164].

During ZEA metabolism, its 6-keto group is reduced, leading to the formation of α-zearalenol (α-ZEL) and β-zearalenol (β-ZEL); upon additional reduction, this leads to the formation of α-zearalanol (α-ZAL) and β-zearalanol (β-ZAL), all of which can be partially conjugated to glucuronic or sulfonic acid (e.g., ZEA-14-O-β-glucuronide, ZEA-14-GlcA) and then excreted in the urine [72,174]. Sometimes, zearalanone (ZAN) is also included in HBM studies [86].

ZEA is the third most frequently included mycotoxin in studies on human urine samples, after OTA and DON (Figure 4). When ZEA is detected in human urine samples, it is normally found in a small proportion of samples, with a few exceptions where detection frequencies exceed 60%, and sometimes reach up to 100% (Table 4). For example, 100% of the analyzed human urine samples were ZEA positive in a study from Germany (with a LOQ set at 0.005 ng/mL) for a cohort of 17 mill workers [132], and the same situation was observed in a study from Hungary (with a LOQ set at 0.003 ng/mL) after analyzing 19 urine samples from coeliac patients [148]. Also, high percentages of ZEA-positive samples were observed in studies from Nigeria (81.7%) [136], China (61.4% [137] and 87.9% [143]), France (66.7%) [147], Hungary (97.6%) [148], and the USA (63%) [170].

Regarding the LOQ, values below 0.005 ng/mL help in the highly sensitive detection of ZEA, providing a more accurate assessment of the population’s exposure to this mycotoxin. ZEA concentrations varied depending on the population and analytical LOQ, ranging from sub-ng/mL levels (such as 0.011–0.174 ng/mL in Hungary [148]) to markedly higher maximum levels, such as 398 ng/mL in rural Nigerian adults [159], and 19.99 ng/mL in another Nigerian cohort [136].

Among the reduced metabolites, α-ZEL and β-ZEL were reported less consistently than ZEA, with detection frequencies spanning 0% (in several studies, including cohorts from Zimbabwe [153] and Bangladesh [94]) up to 82.9% (α-ZEL, Hungary) [148], with concentrations reaching as high as 38.2 ng/mL (α-ZEL, Nigeria) [159] and 31.3 ng/mL (β-ZEL, Chile) [152].

Conjugated forms such as ZEA-14-GlcA and ZAN were measured in fewer studies and generally showed lower or null detection, though exceptions occurred, such as ZEA-14-GlcA in Nigeria (6.7%, with a maximum level of 44.5 ng/mL) [98] and in Portugal (16%, with a maximum level of 25.7 ng/mL) [91]. Other minor metabolites (α-ZAL, β-ZAL, ZAN-14-GlcA, sulfate and glucuronide conjugates) were largely not detected across most cohorts, reflecting either true low prevalence or LOQs too high to capture typical exposure levels.

### 4.4. Trichothecenes

Trichothecenes are a group of tetracyclic sesquiterpenoid chemical compounds, posing a 12,13-epoxytrichothec-9-ene skeleton, a double bond between C-9 and C-10, an epoxide between C-12 and C-13 atoms (considered responsible for their toxicity), and a different number of hydroxyl and acetoxy groups [175]. Over time, mycotoxins have been included in this category based on their chemical similarity, totaling approximately 200 substances [176], but recent studies classify some of the trichothecenes as emerging mycotoxins because they are not legislated, and not routinely determined in mycotoxin monitoring studies [14]. Thus, in this subchapter, DON, HT-2 and T-2 will be presented, and other trichothecenes will be included in the subchapter covering emerging mycotoxins.

#### 4.4.1. Deoxynivalenol (DON)

**Figure 8:**
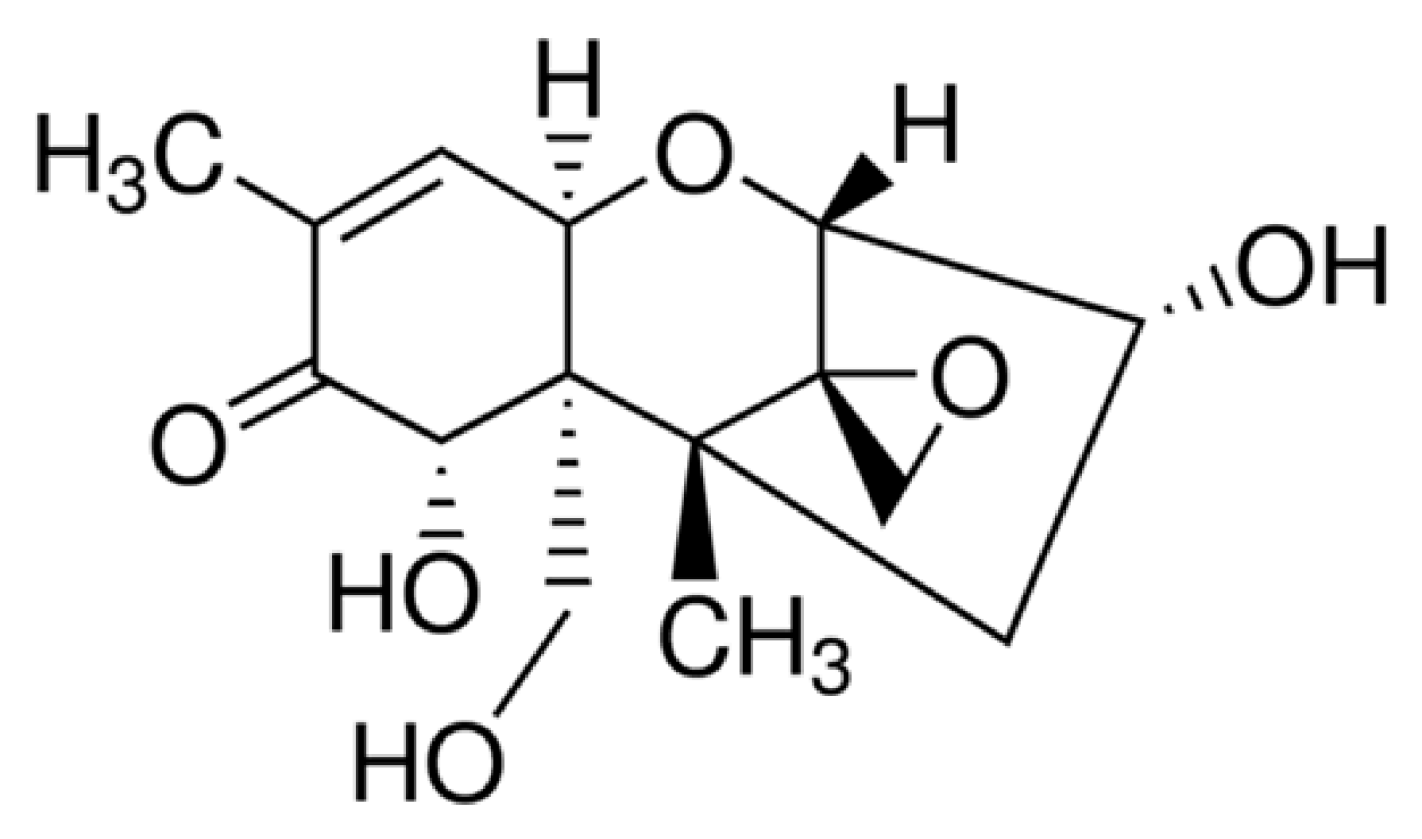
Chemical structure of deoxynivalenol.

DON (Figure 8), known as vomitoxin, with its chemical name 3α,7α,15-trihydroxy-12,13-epoxytrichothec-9-en-8-one, is one of the most studied trichothecenes. The occurrence of DON is linked with the presence of *Fusarium graminearum* and *F. culmorum,* its development being favored by a temperature between 26 °C and 30 °C and an a_w_ of around 0.995 [36]. DON is most frequently found in cereals (corn, wheat, barley), cereal-based products, and animal-based foods such as eggs, organs (kidney), and milk [23].

DON is also named vomitoxin due to its powerful emetic symptoms after consumption, because it is transported into the brain, where it activates dopaminergic receptors. DON, like other trichothecenes, targets the 60S ribosomal subunit, with translational inhibition (inhibition of protein, RNA and DNA synthesis) being the explanation for the toxicity mechanisms. After in vivo and in vitro studies, it was demonstrated that DON can cause damage to organelles such as ribosomes, the endoplasmic reticulum and mitochondria; renal cell apoptosis; rupture of small intestinal villi; impairment of intestinal barrier function; and imbalance of intestinal microbiota, inducing oxidative stress, renal dysfunction, hepatic fat gain, hyperemia, swelling, and apoptosis of mouse endometrial stromal cells, as well as inhibition of cell mitosis and apoptosis of immune cells. Consequently, at the cellular level, DON toxicity can be divided into neurotoxicity, immunotoxicity, and cytotoxicity, while at the organ and tissue level, toxicity can be separated into four blocks, intestinal, hepatic, renal, and reproductive [177]. Even though DON can cause numerous toxic effects upon chronic exposure, the IARC has classified DON as Group 3, “not classifiable as to its carcinogenicity to humans” [164].

DON is one of the main contaminants in food and feed, frequently found along with its acetyl derivatives, 3-acetyl-DON (3-AcDON) and 15-acetyl DON (15-AcDON). When consumed by humans, DON is partially converted to its deepoxy form (deepoxy-DON, also called DOM-1 in the literature), and its metabolism also results in conjugated forms with glucuronic or sulfonic acid, such as DON-3-O-β-glucuronide (DON-3-GlcA), DON-15-O-β-glucuronide (DON-15-GlcA), DON-O-glucuronide (DON-7-GlcA), or DON-3-sulfate, which are then excreted through the kidneys [23,52].

DON is the second most frequently included mycotoxin in studies on human urine samples, after OTA (Figure 4), having been included in nearly half (48%) of the studies aimed at evaluating mycotoxin biomarkers in this matrix. Similar to OTA, the interest in assessing DON in biological matrices—human urine, in this case—is most likely related to the conclusive evidence of its toxicity in animals and humans, as well as its frequent occurrence in both feed and food. Evaluating DON and its metabolites in human urine from over 20 countries, DON was the most frequently measured biomarker (Table 5), with detection rates varying widely: some cohorts showed 0% positivity (e.g., Austrian cereal-restricted diet [121], Iranian esophageal cancer patients [146]), while many others showed near-universal detection, including China (100%) [102], Germany (99–100% in mill workers [132] and 24-h urine samples [154]), Norway (99.6%) [150], France (93.3–100%) [147,155], and Poland (99.5%) [155]. LOQs differed substantially between studies, from as low as 0.015 ng/mL [150] to 35 ng/mL [120], which limits the direct comparability of positivity rates and concentrations across cohorts. DON concentration ranges (in ng/mL) in human urine were also highly variable, ranging from single digits in low-exposure populations to maximum values above 100 ng/mL in several studies, including 436 ng/mL in pregnant US women [170], 140.9 ng/mL in UK children [138], 136 ng/mL in Swedish adolescents [95], and 133.2 ng/mL in Poland [155].

Metabolites such as DOM-1, DON-3-GlcA, DON-15-GlcA, 3-AcDON and 15-AcDON were reported less consistently and generally at lower detection frequencies than DON itself, with several studies reporting them as not detected, though exceptions exist, such as DON-15-GlcA in Portugal (51% positive, maximum value of 204.17 ng/mL) [91] and China (43.8% positive, maximum concentration of 37.7 ng/mL) [139] or DOM-1 in Germany (58.8% positive, maximum level of 0.22 ng/mL) [132].

#### 4.4.2. T-2 Toxin (T-2) and HT-2 Toxin (HT-2)

**Figure 9:**
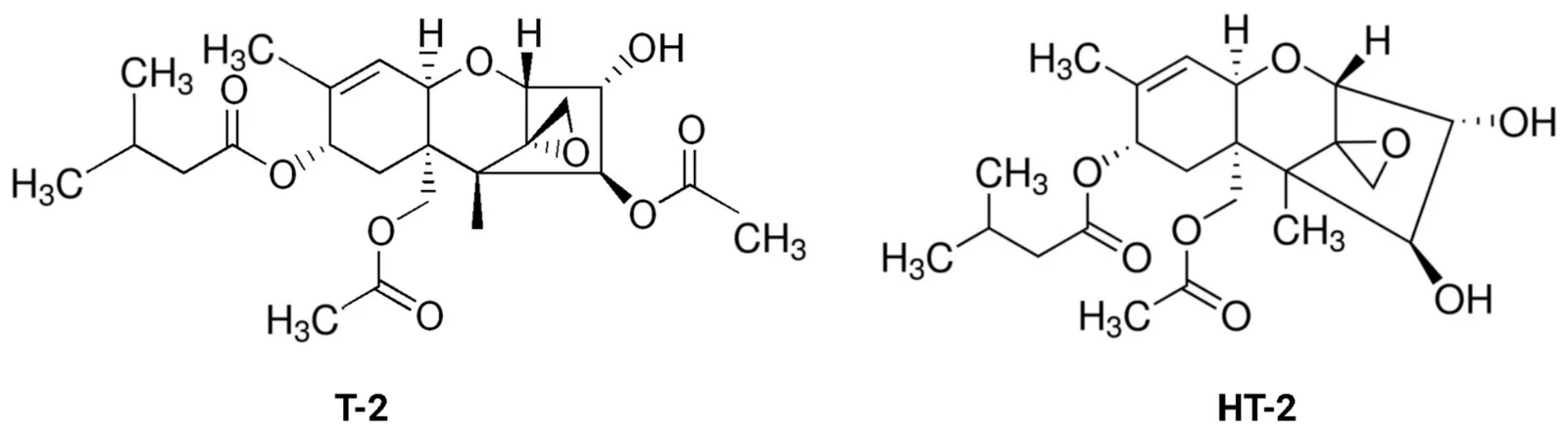
Chemical structure of T-2 and HT-2 toxins.

The T-2 and HT-2 toxins are produced by various *Fusarium* species, particularly *Fusarium sporotrichioides*, *F. poae*, *F. langsethiae*, *F. equiseti*, *F. acuminatum*, *F. moniliforme* and *F. nivale* [36,178]. Chemically, T-2 is (2α,3α,4β,8α)-4,15-bis(acetyloxy)-3-hydroxy-12,13-epoxytrichothec-9-en-8-yl 3-methylbutanoate, while HT-2 is its deacetylated form, being the major metabolite of T-2 (Figure 9). Temperatures between 10 and 30 °C and an a_w_ ranging from 0.93 to 0.995 provide optimum conditions for the growth of fungi that can produce T-2 toxins [23,40].

T-2 and HT-2 are mostly found in cereals (wheat, oats, rye, and barley) and cereal-based products [23]. Like other trichothecenes, they can interfere with protein, RNA and DNA synthesis, the toxic effects of T-2 and HT-2 including growth retardation, myelotoxicity, hematotoxicity, and necrotic lesions on contact sites [179].

Although T-2 and HT-2 have traditionally been assessed as free compounds in human urine, over the past few years, researchers have incorporated their glucuronidated metabolites, such as HT-2-3-glucuronide (HT-2-3-GlcA) and HT-2-4-glucuronide (HT-2-4-GlcA), into HBM investigations [94,130,180,181]. However, this approach has created difficulties regarding the reproducibility of these conjugated compounds during in-house validation steps [130]. On the other hand, recent biomonitoring of T-2, T-2-3-glucoside and their metabolites in human urine—including HT-2-4-GlcA and other phase I and phase II metabolites—using UHPLC coupled to Q-Orbitrap HRMS has revealed a low relevance of conjugated metabolites (0.3% prevalence in 300 human urine samples collected in South Italy) [182], consistent with findings by Gerding et al. [128], who did not detect any HT-2-4-GlcA-positive samples.

Interest in studying T-2 and HT-2 toxins in human urine samples appears to be lower, as they were included in only 27% of the studies covered in this review, ranking after OTA, DON, ZEA, AFs, and FUMOs (Figure 4). This may also be explained by the fact that, in the studies where they were included, T-2 and HT-2 toxins were not detected in most cohorts (Table 6). Detectable levels were reported in a smaller number of studies.

T-2 was found in 2.3% of Chinese adults (ranging from 0.248 to 3.61 ng/mL) [139], 5.8% of Iranian patients with esophageal cancer (maximum of 50.09 ng/mL) [146], 0.5% of the Qatari cohort (but unquantifiable) [149], and 10% of pregnant women in Bangladesh (unquantifiable) [94].

HT-2 exhibited higher positivity rates only in a few specific populations, notably 85% among Norwegian adults (range: 0.078–0.476 ng/mL) [158] and 17.6% among Iranian patients with esophageal cancer (range: 18.91–50.38 ng/mL) [146], while remaining undetected in most other cohorts, including all Bangladeshi samples analyzed for HT-2 conjugates (HT-2-3-GlcA and HT-2-4-GlcA) [94].

### 4.5. Fumonisins (FUMOs)

**Figure 10:**
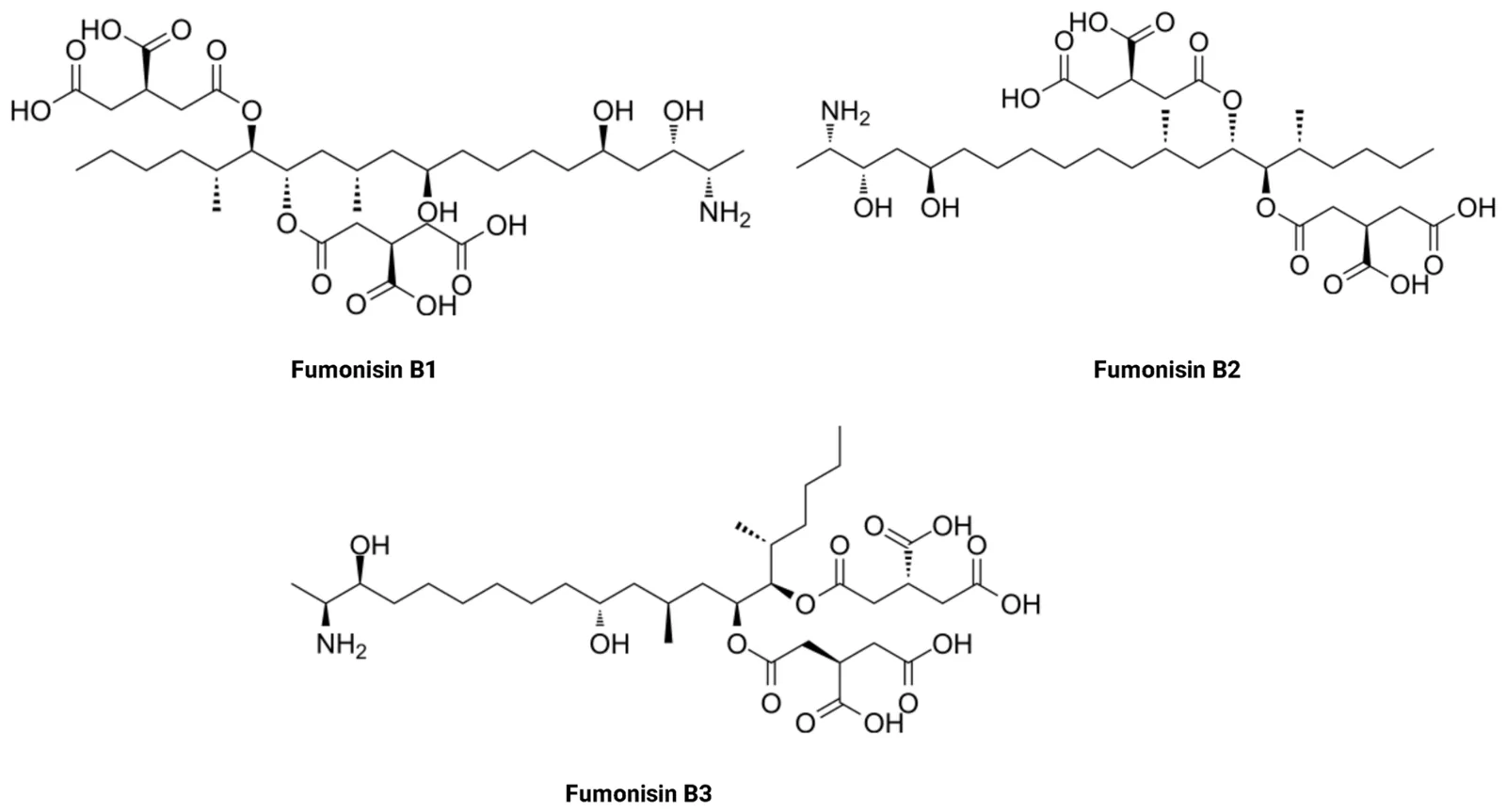
Chemical structure of fumonisins.

FUMOs, FB1, fumonisin B2 (FB2), and fumonisin B3 (FB3) (Figure 10) are produced in most cases by *Fusarium proliferatum*, *F. nygamai*, and *F. oxysporum*, but other species, such as *F. anthophilum*, *F. moniliforme*, *F. dlamini*, *F. globosum*, *F. napiforme*, *F. verticillioides*, and *Alternaria alternate*, can also be considered as FUMO-producing fungi [23,40]. Temperatures between 15 °C and 30 °C and an a_w_ between 0.9 and 0.995 are favorable conditions for the appearance of FUMOs [36]. FB1, the most dangerous and potent of the FUMOs, commonly contaminates maize kernels, but, generally, FUMOs can also be found in sorghum, wheat, barley, soybean, asparagus spears, figs, black tea, and medicinal plants [8].

FUMOs can inhibit sphingolipid synthesis and are linked to esophageal and liver cancers in people. Other in vivo or animal studies pointed out additional negative effects of FUMOs, including neural tube defects, respiratory inflammation, and toxicity in the liver, kidneys, brain (in horses), and lungs (in pigs). In pigs, FUMOs have also been shown to disrupt intestinal integrity, with the effects being more pronounced when FUMOs are co-administered with DON, suggesting a synergistic or additive interaction between the two mycotoxins on gut morphology and function [23,72]. Thus, FB1 and FB2 are classified by the IARC as possible carcinogens (Group 2B), indicating that they may contribute to the growth of cancers in humans [164].

FUMOs rank last among the top 5 most studied mycotoxin groups in human urine samples, after OTA, DON, ZEA and AFs (Figure 4). When FB1 is metabolized, hydrolyzed fumonisin B1 (HFB1) is generated [183,184]. These two forms are the main biomarkers important for inclusion in HBM studies of mycotoxins. Table 7 summarizes urinary FUMO biomarkers (mainly FB1 and FB2, with occasional FB3 and HFB1) reported in approximately 20 human studies. FUMO LOQs varied considerably between studies, ranging from 0.005 ng/mL (FB1) [94] to 15 ng/mL (FB1, FB2) [120]. Based on the reported results, LOQs may partly explain the differences in positivity rates observed across cohorts. FB1 detection rates varied widely, from 0% in several cohorts (urban and rural areas of Portugal [115], Korea [116], Spain [120], Belgium [125], France [147], Sweden [95], Bangladesh [94]) to 100% in Hungarian healthy volunteers and patients with celiac disease (range: 0.141–1.525 ng/mL) [148] and in Zimbabwean adults (range: 0.032–4.65 ng/mL) [153]. High positivity rates were also observed in rural Nigerian adults (71.3%, range: 0.78–566 ng/mL) [159] and in a separate Nigerian cohort (70.8%, range: 0.08–14.88 ng/mL) [136], while Mexican women showed 75% FB1 positivity (range: 0.02–9.312 ng/mL) [111]. In Brazilian pediatric and adolescent groups, both FB1 and FB2 were detected at moderate frequencies (7–48%).

Overall, FUMO concentrations varied from sub-ng/mL levels in Hungary (0.008–1.525 ng/mL for FB1 and FB2) [148] to much higher maximum concentrations, such as 566 ng/mL in rural Nigeria [159] and 12.8 ng/mL in another Nigerian cohort [98]. FB2 was generally detected less frequently than FB1, although it also reached 100% positivity in the Hungarian cohorts, with concentrations up to 0.875 ng/mL [148].

FB3 and HFB1 were reported in only a few studies (Portugal [91] and Belgium [125]) and were consistently not detected (Table 7).

### 4.6. Patulin (PAT)

**Figure 11:**
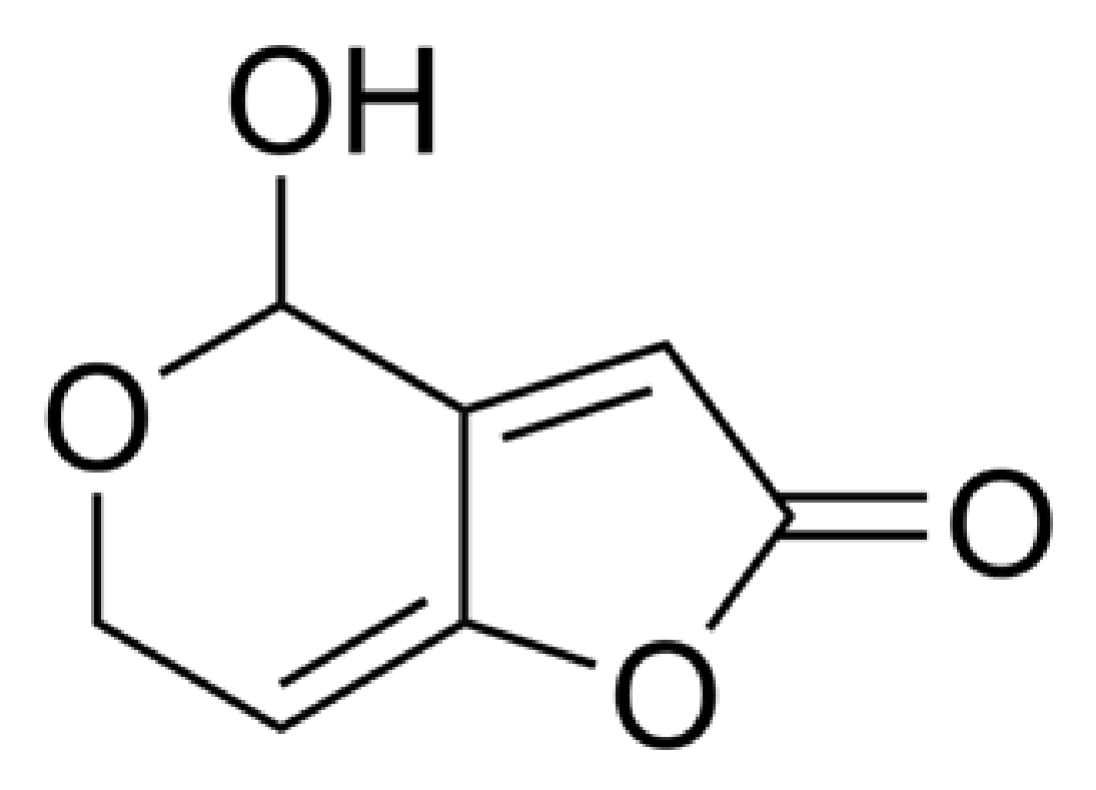
Chemical structure of patulin.

PAT (Figure 11) is a furopyranone, 4-hydroxy-4H-furo[3,2-c]pyran-2(6H)-one, and can be produced by certain species of *Penicillium*, *Aspergillus*, and *Byssochlamys*, with *Penicillium expansum* recognized as the fungus most responsible for its occurrence, followed by *P. crustosum*, *P. patulum*, *P. urticae*, and *P. griseofulvum* [8,23,40]. Its development is favored by temperatures between 16 °C and 35 °C and an a_w_ ranging from 0.83 to 0.96 [36]. PAT predominantly contaminates apples, apple juice, and apple products, but various levels of PAT have also been detected in other foodstuffs such as cereals, legumes, seeds, fruits, nuts, and vegetables [23].

Ingestion of PAT can produce degeneration of epithelial cells, hyperemia and numerous adverse effects in various systems: gastrointestinal (including inflammation of the intestines, distension of the gastrointestinal tract, emesis, intestinal hemorrhage, and ulceration), nervous (agitation, convulsions), respiratory (dyspnea, pulmonary congestion, edema), and nephrotic (damage to the kidney tissues). PAT toxicity can be divided into neurotoxicity, immunotoxicity, genotoxicity, teratogenicity and carcinogenicity [185].

The metabolism of PAT is still not fully understood, which may account for researchers’ reluctance to include this mycotoxin in exposure monitoring studies based on human urine analysis. As a result, only 2 of the 73 studies evaluated between 2000 and 2025 included PAT (Figure 4). When PAT was analyzed in human urine as a free compound, no values [91,144] were detected. The absence of quantifiable PAT in human urine (Table 8) may be due to several reasons, such as low levels of unmetabolized PAT excreted in urine, rapid renal excretion (failing to be collected in the morning urine, which is the most commonly used for the analysis of mycotoxin metabolites), or unknown PAT metabolites that were not analyzed [144].

### 4.7. Citrinin (CIT)

**Figure 12:**
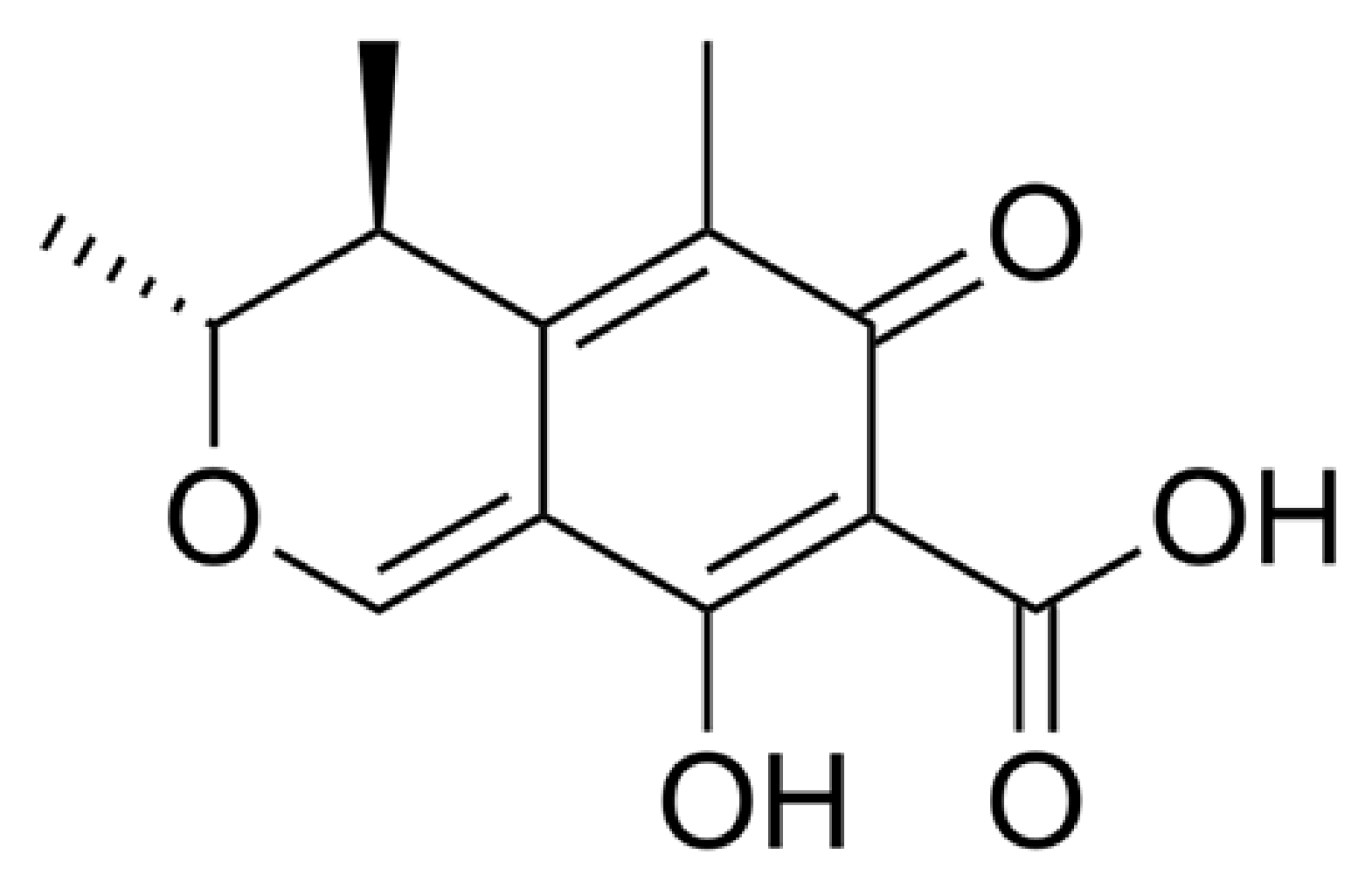
Chemical structure of citrinin.

CIT is a benzopyran derivative (chemically named [3R-trans]-4,6-dihydro-8-hydroxy-3,4,5-trimethyl-6-oxo-3H-2-benzopyran-7-carboxylic acid) (Figure 12) that is produced mostly by *Aspergillus carneus* and *Penicillium citrinum*, when a temperature between 20 °C and 30 °C and an a_w_ from 0.75 to 0.85 are achieved [36]. Other species, such as *P. expansum*, *P. radicicola*, *P. verrucosum*, *Monascus purpureus*, and *M. ruber*, are also considered CIT-producing fungi [40]. CIT was detected in cereals (barley, rice, wheat, oats, maize, and rye), cereal-based products, cheese, sake, red pigments, and spices, as well as foods colored using the *Monascus* pigment [40,72].

CIT interferes with mitochondrial metabolism through enzymatic inhibition; it can accelerate oxidative stress and apoptotic processes and reduce total cell counts. Due to its mechanism of action, CIT can affect all the main organs, including the bone marrow, liver, kidney, and mitochondrial respiratory chain [186].

Humans primarily excrete unmetabolized CIT, along with various amounts of dihydrocitrinone (DH-CIT, also abbreviated in the literature as OH-CIT or HO-CIT) [187].

Unlike PAT, the fact that part of CIT’s metabolism has been elucidated has allowed both CIT and its metabolite DH-CIT to be measured in human urine across a wide range of populations (Table 9), appearing in 15 of the 73 studies from the last quarter-century covered in the present review (Figure 4). Detection rates for CIT varied from 0% (Sweden [95] and Spain [96]) to 100% (German mill workers) [132]. High positivity rates were also reported in several cohorts, including Czech patients with kidney cancer (90%) [140], rural Nigerian adults (37.4%) [159], Tunisian volunteers (72%) [144], and Tunisian patients with colorectal cancer (76%) [144], whereas lower positivity rates were observed in Belgium (2.5%) [125], Qatar (1.1%) [149], and Portugal (1%) [91]. CIT concentration ranges varied considerably and, in some cases, reached very high values, most notably up to 241.46 ng/mL in one Nigerian cohort [136] and 425 ng/mL in another Nigerian cohort [159]. These findings contrasted with much lower maximum concentrations, such as 0.076 ng/mL in German mill workers [132] and 0.087 ng/mL in a Czech cohort [140].

DH-CIT was generally detected at frequencies similar to or higher than those of CIT within the same studies, reaching 100% positivity in both German mill workers [132] and the Czech cohort [140], with concentrations up to 190 ng/mL in rural Nigeria (where the highest CIT concentration was also reported) [159] and 66 ng/mL in pregnant women in the USA [170]. Interestingly, in Bangladeshi pregnant women, DH-CIT was detected more frequently than CIT (78% vs. 40%) [94], with a similar pattern observed in rural Nigerian adults (60.1% vs. 37.4%) [159].

LOQs also varied substantially, ranging from 0.01 ng/mL [136] to 5.76 ng/mL [125] and from 0.01 ng/mL [136] to 0.6 ng/mL [170] for CIT and DH-CIT, respectively. As observed for other mycotoxins, differences in LOQs may partly explain the variability in positivity rates across cohorts.

### 4.8. Emerging Mycotoxins

**Figure 13:**
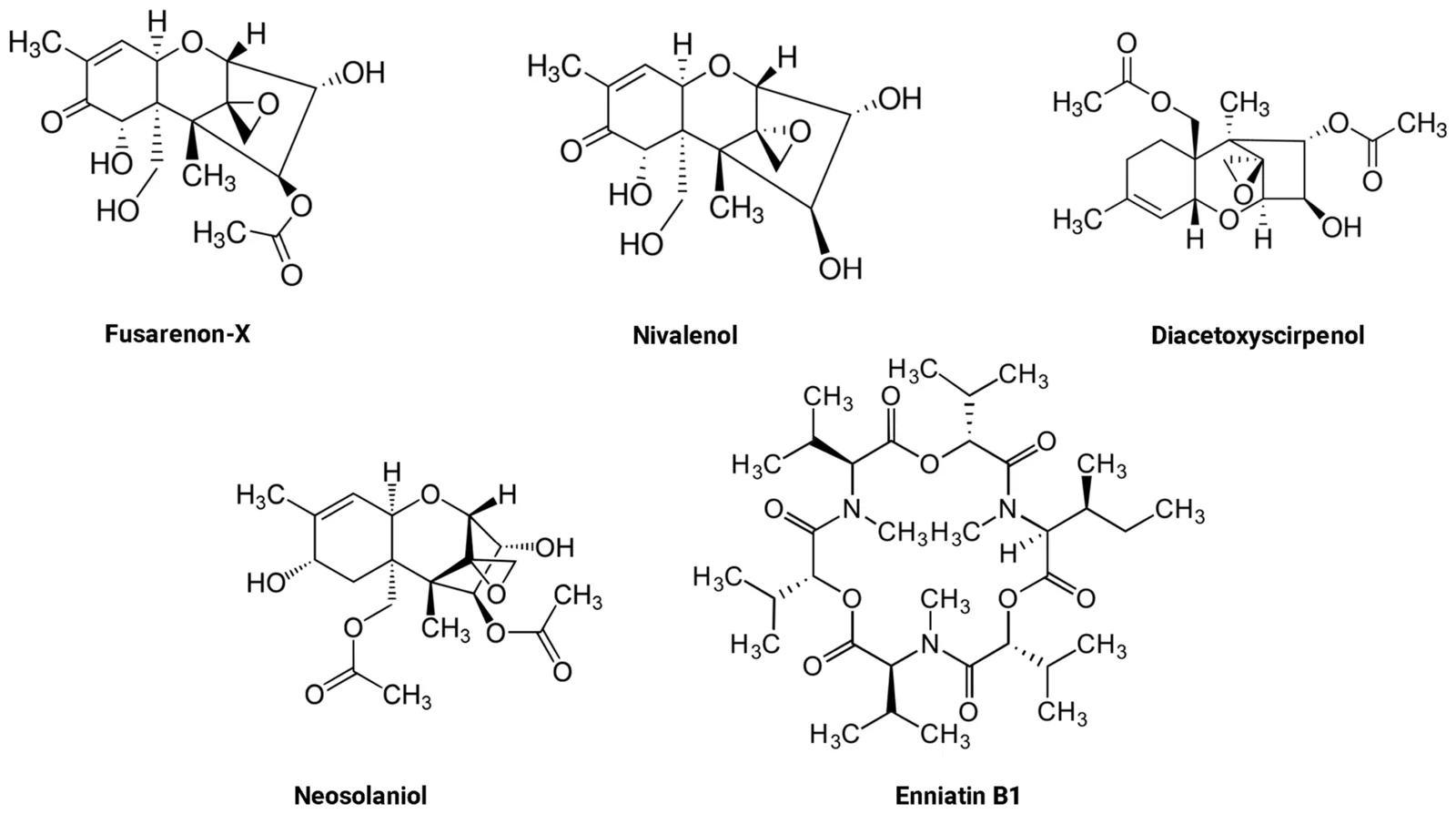
Chemical structures of selected emerging mycotoxins.

Since their discovery, traditional mycotoxins have consistently been investigated, but more researchers have also tried to clarify the issue of emerging mycotoxins concerning their occurrence and toxicity. According to the most recent data, the term “emerging mycotoxins” refers to mycotoxins that are neither routinely determined nor legislatively regulated (Figure 13), including fusaric acid, ENs, BEA, DAS, MON, NIV, STG, culmorin, apicidin, butenolide, fusaproliferin, *Alternaria* toxins, aurofusarin, and emodin [14].

NIV, DAS, NEO, and fusarenone-X (FUS-X) are trichothecenes produced by the *Fusarium* genus, found mostly in grains (such as wheat, maize, rice, millet, sorghum, rye, oats, and barley), coffee, mangoes, soybean, and legumes [72,176]. The major adverse effects of DAS are hematotoxicity and emesis, while FUS-X has been shown to be cytotoxic, teratogenic, and carcinogenic. Like other trichothecenes, NIV induces alterations in DNA synthesis, affecting organs and tissues such as the colon, jejunum, stomach, bone marrow, and kidney [72].

STG can be produced by *Aspergillus flavus* and *A. versicolor*, with its development favored by temperatures between 5 °C and 25 °C and an a_w_ ranging from 0.8 to 0.95 [36]. STG has been detected in grains and grain-based products, coffee, spices, nuts, beer, and cheese. There are significant structural and biological similarities between AFB1 and STG; it is recognized that STG can act as a biogenic precursor of AFB1. Epidemiological studies have revealed that STG can be characterized by carcinogenicity, its presence being linked to gastric carcinoma, hepatocellular carcinoma, and liver cirrhosis in animals [188]. The IARC classified STG as a Group 2B carcinogen, which means that it is possibly carcinogenic to humans [164].

ENs are produced mostly by the *Fusarium*, *Alternaria*, *Halosarpheia*, and *Verticillium* genera [189], and some of them (particularly ENs A, A1, B, B1–ENA, ENA1, ENB, ENB1, respectively) have been detected in cereals and cereal by-products [190]. Some studies gave proof of the potential cytotoxicity and genotoxicity of ENs [189], without any regulation in this context.

*Alternaria* fungi are plant pathogens capable of adapting to various environmental conditions. Their spores can infect plants and germinate across a broad temperature range, from 4 to 35 °C. The scientific community has increasingly focused on studying *Alternaria*-derived toxins in recent years, the most extensively studied being alternariol monomethyl ether (AME) and alternariol (AOH). These toxins are commonly detected in grains, fruits, and fruit-derived products, as well as in legumes, nuts, tomatoes, and oilseed-based foods. Like other mycotoxins, *Alternaria* toxins can pose significant health risks to humans, including mutagenic and genotoxic effects, esophageal cancer, and disruption of endocrine function [191].

Emerging mycotoxins are quantified in human urine as free compounds, probably due to the lack of analytical reference standards for metabolites, but also due to the scarce information regarding their metabolism. Only a small number of studies (under 15%) included emerging mycotoxins in human urine analysis (Figure 4), most often within multi-component studies (Table 1), alongside mycotoxins with known metabolism and demonstrated quantifiable presence. Detection rates of emerging mycotoxins differed substantially among studies, a variation attributable to differing LOQs. For example, NIV was quantified in 33.3% of the subjects in a Nigerian study [136], and in 2.4% of the healthy participants included in a study from Hungary [148], with a maximum concentration of 3.02 ng/mL [136]. NEO was detected in another study from Iran, in healthy volunteers and patients with esophageal cancer, with frequencies of 40% and 5.8%, respectively. The next-generation biomonitoring LC-MS/MS study by Feuerstein et al. involving 446 pregnant Bangladeshi women detected mycoestrogens AOH and AME in 8.9% and 34% of samples, respectively [160] (Table 10).

## 5. Mycotoxin Exposure Assessment Using Human Urine Analysis

Human mycotoxin-related risks are influenced by dose and hazard. To reduce human health risks, preventive measures against mycotoxin contamination must be established. Good farming and storage practices, regulated MPLs, pollution management, and continuous exposure assessment are all included in this action plan. Studies on mycotoxin exposure evaluation contribute significantly to risk assessment and risk management, as well as to the establishment of legal regulations for mycotoxin monitoring and control in the food chain [35].

Considering that mycotoxins are present in a wide variety of food products that are consumed worldwide, as well as through exposure via inhalation, it is essential to establish reliable strategies to assess human risk. According to FAO/WHO CAC and the National Research Council (NCR) of the USA, risk analysis is a food safety approach with three blocks of information:

- risk assessment (a thorough examination of the prevalence of known or possible harmful health effects from dietary risks in humans or animals),
- risk management (a process of scaling policy alternatives considering risk assessment and strategies of surveillance and regulatory responses),
- risk communication (the dissemination of information regarding risk management alternatives and strategies among risk managers, consumers and other interested parties: prioritizations, decisions, actions, guidelines, monitoring design) [192,193,194].

All decisions should pursue accessible scientific knowledge, expert awareness, and strength of the evidence. Moreover, a recent publication by Smaoui et al. [43] clearly underlines the importance of scientific research in this entire process of assessing the risk of exposure to mycotoxins, including directions such as: multiresidue analysis; toxicological studies; omics studies; absorption, distribution, metabolism, and excretion (ADME); and masked mycotoxins.

Human health risk assessment should comprise four steps: hazard identification, hazard characterization, exposure assessment, and risk characterization, all based only on scientifically relevant data (data from both animal and human studies) and on realistic exposure scenarios [193,194]. Estimating the frequency, severity, and duration of mycotoxin consumption is necessary for an exposure evaluation. On the other hand, risk characterization involves determining the level of concern by comparing the exposure assessment results with the hazard characterization [74].

Mycotoxin exposure assessment is a complex and difficult approach, with many critical points that must be considered, requiring data on food consumption, data on mycotoxin concentrations in food and/or data on mycotoxin levels in biological samples. To perform a dietary mycotoxin exposure assessment in humans, data on mycotoxins in foods (levels and frequency) is often combined with data concerning food consumption, following either a deterministic or probabilistic approach [80,195,196]. To assess food consumption, data can be collected from:

- food consumption surveys,
- food monitoring studies,
- total diet studies,
- duplicate diet studies [80].

Deterministic estimations of exposure make the assumption that the parent mycotoxins and their masked forms are continuously present at an average level, and that all people consume specific food groups at the same time and at the same level. However, in probabilistic approaches (such as Monte Carlo simulation), all possible values for each variable are considered, obtaining all plausible combinations of daily consumptions and mycotoxin levels in a population group, reflecting a probability distribution of exposure values and providing a more complex assessment of the likelihood of chemical exposure [80,195,197].

After these traditional (deterministic or probabilistic) approaches, various parameters can be calculated: the intake level in one day, normally named estimated daily intake (EDI) and expressed in ng/kg body weight (bw)/day; the benchmark dose defined as a 10% increase over the background, named BMDL10 and expressed in ng/kg bw/day; the margin of exposure (MOE) calculated as the ratio between BMDL10 and EDI; the margin of safety (MOS) calculated as the ratio between EDI and the tolerable daily intake (TDI) [197]. TDI levels or acceptable daily intakes (ADI) for humans are health-based guidance values derived from the no-observed-adverse-effect levels (NOAEL) based on research on animals. A TDI set is an estimation of the daily amount of a pollutant in food, drinking water, and air that can be taken in for a lifetime without posing a significant risk to health [74].

Based on reliable toxicological data, for the regulated mycotoxins, TDIs or provisional maximum TDIs (PMTDIs) have been established. The JECFA, SCF, and EFSA set TDI values for DON (1 μg/kg bw/day) [198], and ZEA (0.25 μg/kg bw/day) [199], and PMTDIs for NIV (0.7 μg/kg bw/day) [198], the sum of DON, 3AcDON and 15AcDON (1 μg/kg bw/day) [200], and the sum of T-2 and HT-2 (0.1 μg/kg bw/day) [201], DAS (0.65 μg/kg bw/day) [202] and PAT (0.4 μg/kg bw/day) [203]. For FUMOs, the JECFA proposed a PMTDI of 2 μg/kg bw/day (FB1, FB2 and FB3 alone or combined) [204], but in 2018, the EFSA established a TDI for FB1 alone (1 μg/kg bw/day) [205]. As AFs are classified as carcinogenic to humans (IARC Group 1), there is no TDI proposed; their levels in food are recommended to be as low as reasonably achievable (ALARA) [206], while the EFSA recommends using a MOE value of 10,000 (ten thousand) or higher for minimal concern regarding AFs and their public health consequences [207]. In the same way, because OTA was classified as potentially carcinogenic to humans (IARC Group 2B), the EFSA suggested applying a MOE approach to characterize its risk [208].

Most of the risk assessments concerning non-carcinogenic mycotoxins characterize risk through a straightforward comparison of the EDI against established health-based guidance values, such as the TDI or PMTDI, which are defined for individual mycotoxins. This approach is methodologically equivalent to the Hazard Quotient (HQ) framework, in which the HQ is calculated as the ratio of the mycotoxin EDI to its corresponding health-based guidance value. An HQ below 1 indicates a negligible risk to human health, whereas an HQ higher than 1 suggests a potential concern for adverse health effects in the exposed population [209].

Mycotoxin exposure assessment determined based on hazard quantification in food and food consumption data presents some disadvantages, such as difficulty in selecting a representative number of food samples, large amounts of data to be processed, and exposure assessment based only on oral ingestion. Thus, the measurement of specific urinary mycotoxin biomarkers can be a reliable alternative to evaluate mycotoxin exposure, with advantages including extended exposure measurement (orally or by inhalation), more accurate estimation of the risk, individual-level information, no need for consumption data collection, and a powerful tool that avoids misleading sampling [80,210].

As mentioned in Section 2, HBM needs validated biomarkers correlated with mycotoxin metabolism and kinetics in humans. These biomarkers should be characterized by plausibility, dose–response, time-response, robustness, reliability, stability, analytical performance (such as precision and accuracy), and reproducibility [211].

The exposure assessment determined based on urinary biomarkers needs to include in its design the toxicokinetic data, considering the urinary excretion ratio of each mycotoxin with the aim of acquiring the most representative and rational exposure assessment scenarios. When urinary data is used, a probable daily intake (PDI) is calculated according to the following formula:

$$\mathrm{PDI}\;(µg/\mathrm{kg}\;\mathrm{bw}/\mathrm{day})=\frac{C\times V}{W\times E}\times 100$$

where C refers to the average value of mycotoxin biomarker concentrations in urine (µg/L), V is the volume of urine excreted in 24 h, W refers to body weight (kg), while E represents the mycotoxin excretion rate (%). To have different mycotoxin exposure scenarios, and according to the EFSA recommendation [212], three different ways for C value calculation can be used: (1) the mean of urinary mycotoxin levels considering only the positive samples (above LOQ); (2) the lower bound (LB) scenario, when zero is assigned to samples in which mycotoxins are not detected or are detected between LOD and LOQ, this being the less conservative scenario, known as the optimistic approach; (3) the upper bound (UB) scenario, when LOD is assumed for the samples ≤ LOD and the LOQ value for the samples between LOD and LOQ, this being the most conservative scenario, known as the pessimistic approach. Sometimes, this equation also includes a correction of the mycotoxin concentrations using the creatinine index, as described by Rodríguez-Carrasco et al. [93], but since no consensus has been reached on this practice—as creatinine can be influenced by various individual factors [81,82]—the step of adjusting the C value by the creatinine ratio is often neglected. The daily urine volume (V) is set in accordance with the literature and the particularities (age) of the sampling group, namely 1.5 L/day for adults, 1.25 L/day for adolescents and 1.0 L/day for children [118]. Other authors proposed a range of values, such as 1–2 mL/kg bw/h for children and 0.5–1 mL/kg bw/h for adolescents [213], which is a useful option if various scenarios regarding the volume of excretion are proposed, as was done for example in a study by Papageorgiou et al. [138] who proposed minimum, maximum, and mean values within the range. For dietary exposure assessment studies, a body weight (W) of 70 kg is used as default for the European adult population (aged 18–64 years), or more specifically, 67.2 kg for females and 82 kg for males, while for children and adolescents specific values are employed (12 kg for toddlers aged 1–3 years, 23 kg for other children aged 3–10 years, 43.4 kg for adolescents aged 10–14 years, and 61.3 kg for adolescents aged 14–18 years), all of these according to the EFSA guidance [214]. In some cases, the body weight used to calculate PDI is provided in the questionnaire filled out by each participant included in the study [92,138]. The urinary mycotoxin excretion rate (E) is replaced with the appropriate values found for humans in the literature: 72% for DON [118], 10% for ZEA [90], 2.5% for OTA [148], 1.3% for AFs [97], and 0.5% for FB1 [215].

During the past ten years, there has been a high interest in calculating the PDI of mycotoxins based on their urinary concentrations. In 2014, Rodríguez-Carrasco et al. [92] found that 8.1% of the total Spanish subjects included in the study (22 males, 16 females, 16 children) were estimated to exceed the DON PMTDI (1 μg/kg bw/day), 51.3% of exposed individuals amounted to between 50% and 99% of the DON PMTDI, and 2 out of 9 exposed children exceeded the safety levels. The potential risk linked with mycotoxin exposure in children and adolescents was demonstrated by Papageorgiou et al. [138], who suggested that 33–63% of children (3–9 years, *n* = 40) and 5–46% of adolescents (10–17 years, *n* = 39) from the United Kingdom exceeded current guidance regarding the PMTDI for DON.

In 2021, the results of a study from Hungary (including 60 subjects, 41 healthy people and 19 coeliac patients) revealed for FB1 a mean PDI of 1.172 μg/kg bw/day in healthy people and 1.116 μg/kg bw/day in coeliac patients, in both situations exceeding the FB1 TDI (1 μg/kg bw/day) [205]. For OTA, high PDI values were also noted: 0.150 μg/kg bw/day in healthy people and 0.092 μg/kg bw/day in coeliac patients (compared with a proposed TDI of 0.017 μg/kg bw/day) [148].

Under the LB and UB scenarios, the PDI values based on urinary biomarkers in Spain were 9.1 µg/kg bw/day and 10.85 µg/kg bw/day, respectively, for AFB2; 15.9 µg/kg bw/day and 17.2 µg/kg bw/day, respectively, for AFG2; and 0.07 µg/kg bw/day and 0.19 µg/kg bw/day, respectively, for OTB, all these values revealing a potential health risk (MoE < 10,000) [87].

In a publication by Namorado et al., under the European Human Biomonitoring Initiative (HBM4EU), based on urinary levels from 1270 participants from six countries (France, Germany, Iceland, Luxembourg, Poland and Portugal), the exposure to total DON was assessed considering the approach using PDI estimation through reverse dosimetry. Results from the multiple linear regression models confirmed the differences concerning the sampling season; for France, a higher risk of exposure to DON was registered in the summer, while in Luxembourg, the highest risk was observed in the spring, and in Portugal during both the spring and the summer. All the participants from Germany and almost all from Iceland presented exposure to DON below the TDI, indicating that there is no health risk associated, while 8.4%, 9.8%, 13.3%, and 17.1% of people in Luxembourg, Portugal, France, and Poland, respectively, presented an HQ greater than one, reflecting an increased likelihood of a toxicological response to DON [155].

In a recent publication from 2025 [157], Ali et al. assessed mycotoxin exposure in Brazilian children (*n* = 104) and reported that, under the UB scenario, the 95th percentile EDI for AFM1 in preschoolers reached 0.786 μg/kg bw/day, far exceeding the BMDL10 threshold, with MoE values below 10,000 in all age groups (preschoolers 3–6 years, schoolers 7–10 years, and adolescents 11–17 years), indicating carcinogenic concern. OTA was identified as the mycotoxin with the highest toxicological concern, with HQ values exceeding unity across the entirety of both the preschool-aged (median HQ: 1.71) and school-aged (median HQ: 1.40) groups. FUMOs exhibited a comparable pattern, with HQ values surpassing unity in a subset of both preschool- and school-aged individuals; however, the overall magnitude of exposure-related risk was comparatively lower than that observed for OTA. Notably, 95th percentile exposure estimates exceeded both the ML and the TDI values established for FUMOs in specific subgroups of the pediatric population. On this basis, the authors concluded that, although risks at the population level appear to be of limited magnitude, exceedances at the individual level cannot be considered negligible and warrant continued attention. For the sum of ZEN equivalents, the 95th percentile EDI exceeded the group TDI of 0.25 μg/kg bw/day in all age groups, with HQ > 1 observed in approximately 77% of preschoolers (median HQ: 1.14).

Ezekiel et al., studying rural adults (*n* = 286) from Nasarawa and Niger (regions in North-central Nigeria), reported average PDI values for FB1 of 990 and 418 μg/kg bw/day, respectively, both dramatically exceeding the TDI of 1 μg/kg bw/day. Average PDI values for ZEN in both states (11 and 4 μg/kg bw/day) exceeded the TDI of 0.25 μg/kg bw/day by 16- to 44-fold, while CIT average PDIs (0.7–3 μg/kg bw/day) exceeded EFSA’s level of no concern for nephrotoxicity (0.2 μg/kg bw/day) by 3- to 20-fold. OTA MoE values for both non-neoplastic and neoplastic endpoints were below 200 and 10,000, respectively, indicating chronic risk in both states. AFB1 MoE values were far below 10,000, with average values of 1.7 and 2.7 in Nasarawa and Niger, confirming significant carcinogenic concern. Also, significant seasonal variability was observed across three mycotoxin groups—AFs, citrinin, and FUMOs—with urinary concentrations and exposure being significantly elevated during the harvest season relative to the storage season among communities in Nasarawa state [159].

The United Arab Emirates AF biomonitoring study by Elabed et al. (2025) [156] did not formally calculate EDI values but detected AFM1 using an immunoassay in 69% of adult urine samples at an overall mean of 0.792 ng/mg creatinine, with males showing higher levels (0.912 ng/mg creatinine) than females (0.676 ng/mg creatinine), and identified a significant correlation between rice consumption and AFM1 levels in males (*p* = 0.038).

Interestingly, McKeon et al. investigated, using a statistical model, the relationship between daily intake and urinary excretion of T-2 and HT-2 toxins in 40 Norwegian adults, estimating that approximately 18.4% of the external exposure could be traced back in urine within 24 h, with a mean daily cumulative T-2 and HT-2 exposure of 8907 ng per person, and an average total HT-2 concentration excreted over 24 h of 0.676 ng/mL. While no formal EDI-to-TDI comparison was provided, the study highlighted that a substantial fraction (around 80%) of ingested T-2 and HT-2 remains unaccounted for in urine, underscoring uncertainties in exposure assessment for these trichothecenes relative to the EFSA group TDI of 0.02 μg/kg bw/day [158].

## 6. Conclusions

Analyzing the data included in the present review, it can be observed that, in the last ten years, there has been increased interest in assessing human exposure to mycotoxins through the analysis of biological samples, particularly urine, due to the ease of sample collection. Complex analytical techniques that simultaneously evaluate mycotoxins alongside other substances, such as pesticides or contaminants, have also been described recently. However, few data are available on the presence of patulin and emerging mycotoxins in human urine, and study design remains limited by the lack of commercial analytical reference standards, particularly when simultaneous analyses and available laboratory equipment are considered. Publications describing environmentally friendly extraction techniques for mycotoxins from human urine have only appeared in the past year, despite this approach having been increasingly promoted over the last decade.

Overall, the data presented in this study underscores the value of biological samples, particularly urine, as a useful tool for assessing human mycotoxin exposure.

As a future perspective, electrochemical aptasensors focused on mycotoxin detection may eventually represent promising candidates for providing rapid data for health monitoring, including the evaluation of mycotoxin exposure through urine. However, this potential application remains at a conceptual and exploratory stage, as no data currently support its feasibility, and substantial further research and validation will be required.

## 7. Materials and Methods

To proceed with a complex evaluation of the scientific data regarding mycotoxin monitoring in human urine as a tool for assessing health risks, this literature review engaged in an in-depth examination of published articles available in databases, including Web of Science, PubMed, Science Direct and Scopus. In order to select the relevant articles, a combination of specific keywords and their synonyms was used, along with Boolean operators to refine the results: (“mycotoxins” OR “aflatoxins” OR “ochratoxin” OR “fumonisin” OR “trichothecenes” OR “deoxynivalenol” OR “HT-2” OR “T-2” OR “zearalenone” OR “patulin” OR “citrinin” OR “emerging mycotoxins” OR “enniatins”) AND (“human urine” OR “urinary excretion” OR “urine biomonitoring” OR “urine”).

In the first step of manuscript preparation, both original research and review papers were included to ensure a comprehensive overview of the mycotoxin biomonitoring process in human urine. Inclusion criteria were: (i) research including mycotoxin evaluation in human urine; (ii) original articles and reviews published in the last 25 years (from 2000 to 2025); (iii) research published in English to guarantee consistent interpretation of the data; (iv) full text available. Exclusion criteria included: (i) studies that analyze mycotoxins in other types of urine, not human urine; (ii) research published in forms other than original research and reviews (e.g., letters to the editor, communications, and abstract proceedings); (iii) research not available *in extenso*; (iv) studies published in languages other than English; (v) research published prior to 2000.

Relevant articles were periodically identified through searches of the mentioned databases over a period of approximately 12 months to ensure continuous and up-to-date documentation across all sections of the paper. The last search of these databases was conducted on April 30, 2026. As the present article was structured as a narrative review, the articles were manually selected and reviewed in full-text version by 2 authors (O.M. and D.P.) and recorded in an in-house database comprising DOI, title, authors, publication year, publication type (review/original article), and useful sections. Duplicates were removed manually based on the unique DOI link. Review studies and book chapters were used exclusively to document the state of the art. In the state of the art, information published prior to 2000 was introduced only if its contribution was considered significant or innovative and no recent data was available. For the *Occurrence* section of the present review, only original research surveys from the in-house database focused on the analysis of mycotoxins in human urine were included (minimum information included: country of the study; sample size; biomarker monitored; number of positive samples/percentage of positive samples or range of the biomarker in ng/mL/maximum value registered mentioned), meaning 73 articles that fulfilled this requirement.

The strengths of this study lie in the large number of original articles included in the review and the synthesis of a wide variety of data, providing a comprehensive analysis of mycotoxin biomonitoring in human urine. Additionally, when the original papers were examined, greater attention was given to the abbreviations used, examining each name. This ensured that a single abbreviation for a term would be used consistently in the current review, minimizing any potential misunderstanding.

However, there are three limitations of the review, including the large period covered (this fact may lead to the possibility that some articles may be omitted due to the large number of data to be processed), the English language filter required, and the lack of mycotoxin concentrations expressed in ng mycotoxin/mL urine in some articles, occasionally creatinine correction being applied with results expressed in ng mycotoxin/mg creatinine.

The comparability of exposure estimates across the studies included in this review is also subject to several methodological constraints. Urinary concentrations are commonly converted into estimated daily intake using standard assumptions about urine output; however, actual urine volume may vary considerably between individuals according to fluid intake, body size, age, and environmental conditions, increasing uncertainty in the resulting exposure estimates. The normalization of urinary concentrations to creatinine introduces further constraints, as this approach relies on the assumption of relatively stable creatinine excretion. In practice, creatinine output can vary with muscle mass, dietary protein intake, kidney function, and other individual characteristics, independently of urine dilution. Moreover, the procedures used for creatinine adjustment are not uniform across studies, while some studies do not apply this correction, limiting the reliability of direct numerical comparisons between reported biomarker concentrations. Analytical sensitivity also imposes constraints on cross-study comparisons, since differences in detection and quantification limits mean that a non-detect may reflect a less sensitive analytical method rather than the true absence of mycotoxin. Overall, these methodological constraints contribute to heterogeneity in exposure estimates and should be considered when interpreting and comparing findings across studies.

## Figures and Tables

**Table 2:** Selected studies on biomarkers of aflatoxins in human urine (presented in ascending order by publication date).

Country	Sample Size (Particularities)	Biomarker *	LOQ (ng/mL)	No. Positive Samples	% Positive Samples	Range (ng/mL)	Ref.
Sierra Leone	134 (boys, dry season)	AFB1	NA	47	35	0.6–188	[99]
		AFM1	NA	56	41	0.5–374	
		AFL	NA	83	62	0.04–14.2	
		AFB2	NA	40	30	0.01–15.5	
		AFM2	NA	71	53	4.5–130	
		AFG1	NA	51	38	2.9–169	
		AFG2	NA	3	2	0.1–1.5	
	110 (girls, dry season)	AFB1	NA	53	49	0.04–319	
		AFM1	NA	48	44	2.3–34	
		AFL	NA	68	62	0.05–8.9	
		AFB2	NA	18	17	0.2–152	
		AFM2	NA	48	44	4.5–94	
		AFG1	NA	42	39	0.4–138	
		AFG2	NA	0	0	nd	
	97 (boys, rainy season)	AFB1	NA	32	33	1.2–115	
		AFM1	NA	42	43	0.1–35	
		AFL	NA	52	54	0.02–7.2	
		AFB2	NA	9	9	0.2–48	
		AFM2	NA	62	64	1.3–41.3	
		AFG1	NA	27	28	0.8–57.4	
		AFG2	NA	2	2	0.2–0.7	
	93 (girls, rainy season)	AFB1	NA	38	41	0.08–127	
		AFM1	NA	55	59	0.3–124	
		AFL	NA	35	38	0.1–9.0	
		AFB2	NA	19	20	0.1–12	
		AFM2	NA	66	71	5.1–86	
		AFG1	NA	18	19	1.0–150	
		AFG2	NA	3	3	1.1–2.0	
Korea	12	AFM1	0.01	1	8.3	0.09	[116]
Spain	27 (healthy volunteers)	AFB1	1.5	0	0	nd	[120]
		AFB2	1.2	0	0	nd	
		AFG1	1.2	0	0	nd	
		AFG2	2	1	4	uq	
Italy	10 (5 female, 5 male)	AFM1	0.12	0	0	nd	[122]
Belgium	40	AFB1	1.66	0	0	nd	[125]
		AFM1	0.02	0	0	nd	
		AFB1-N7-Gua	1.7	0	0	nd	
Nigeria	120 (all ages)	AFM1	0.15	17	14.2	max. 1.5	[98]
Brazil	113	AFM1	0.00025	74	65	NA	[97]
		AFB1-N7-Gua	0.01	0	0	nd	
Nigeria	120 (children, adolescent and adults)	AFM1	0.001	87	72.5	0.001–0.62	[136]
China	260 (adults)	AFB1	0.2	0	0	nd	[139]
		AFB2	0.1	0	0	nd	
		AFG1	0.1	0	0	nd	
		AFG2	0.2	0	0	nd	
		AFB1-lysine	NA	0	0	nd	
		AFM1	0.1	27	10.4	0.125–0.464	
		AFM2	0.2	0	0	nd	
Bangladesh	154 (children and infants)	AFM1	0.005	67	43.5	1.7–75.3	[142]
Pakistan	264	AFM1	0.005	182	69	max. 0.393	[145]
France	9 (occupational exposure)	AFB1	0.01	5	55.5	max. 0.239	[147]
		AFM1	0.05	4	44.4	max. 0.46	
Hungary	41 (healthy people)	AFM1	0.063	0	0	nd	[148]
	19 (coeliac patients)	AFM1	0.063	0	0	nd	
Qatar	559	AFB1	0.2	2	0.4	uq	[149]
		AFB2	0.2	13	2.3	uq	
		AFG2	0.2	9	1.6	0.19–0.34	
		AFM1	0.2	3	0.5	uq	
Chile	172	AFB1	0.1	14	8	max. 0.3	[152]
		AFM1	1.1	2	1	max. 2.2	
Zimbabwe	50	AFM1	0.025	9	18	max. 0.87	[153]
Spain	56 (healthy people)	AFB2	5	18	32	max. 60.98	[87]
		AFG2	5	23	41	max. 69.42	
United Arab Emirates	144	AFM1	NA	100	69	NA	[156]
	(healthy people)						
Brazil	26 (preschoolers)	AFM1	0.03	4	15	NA	[157]
	29 (schoolers)	AFM1	0.03	5	17	NA	
	49 (adolescents)	AFM1	0.03	3	6	NA	
Spain	25	AFB1	0.005	25	100	0.015–0.498	[96]
		AFB2	0.005	25	100	0.009–0.299	
		AFG1	0.005	6	24	max. 0.244	
		AFG2	0.01	25	100	0.369–5.276	
		AFM1	0.01	25	100	0.025–3.415	
Bangladesh	50 (pregnant women)	AFM1	0.06	0	0	nd	[94]
Nigeria	286 (adults—rural areas)	AFM1	0.01	115	40.2	0.10–3.14	[159]

LOQ: limit of quantification; NA: not available; nd: not detected; uq: unquantifiable (higher than the limit of detection, but below the limit of quantification). * Abbreviations defined in the footnote of Table 1.

**Table 3:** Selected studies on biomarkers of ochratoxins in human urine (presented in ascending order by publication date).

Country	Sample size (Particularities)	Biomarker *	LOQ (ng/mL)	No. Positive Samples	% Positive Samples	Range (ng/mL)	Ref.
Sierra Leone	134 (boys, dry season)	OTA	NA	29	21	0.07–59	[99]
		4-OH-OTA	NA	50	37	0.1–29	
		OTB	NA	64	47	0.4–218	
	110 (girls, dry season)	OTA	NA	34	31	0.08–148	
		4-OH-OTA	NA	41	38	0.1–14.7	
		OTB	NA	51	47	0.6–124	
	97 (boys, rainy season)	OTA	NA	26	27	0.6–72.2	
		4-OH-OTA	NA	28	29	0.2–37	
		OTB	NA	31	32	0.05–45	
	93 (girls, rainy season)	OTA	NA	21	23	0.7–4.9	
		4-OH-OTA	NA	48	52	0.2–33	
		OTB	NA	41	44	0.06–81	
Italy	38 (healthy volunteers)	OTA	NA	22	58	0.012–0.046	[100]
	3 (patients with KIN)	OTA	NA	3	100	max. 0.140	
UK	50	OTA	0.01	46	92	0.01–0.058	[101]
Croatia	35 (healthy volunteers)	OTA	0.9	33	94	0.99–5.22	[103]
Hungary	88	OTA	0.006	54	61	0.006–0.065	[104]
Portugal	60	OTA	0.02	42	70	0.02–0.105	[105]
Portugal	30 (morning sampling)	OTA	0.007	13	43.3	0.011–0.208	[109]
	30 (afternoon sampling)	OTA	0.007	14	46.7	0.008–0.110	
Spain	31 (morning sampling)	OTA	0.007	25	80.6	0.007–0.124	[109]
	31 (afternoon sampling)	OTA	0.007	26	83.9	0.008–0.089	
Portugal	43 (healthy volunteers)	OTA	0.008	27	62.8	max. 0.071	[112]
Germany	13 (healthy volunteers)	OTA	0.05	13	100	0.20–0.29	[113]
		OTα	0.05	13	100	0.65–1.64	
Portugal	155 (winter season)	OTA	0.008	122	78.7	max. 0.069	[114]
Korea	12	OTA	0.004	12	100	0.012–0.093	[116]
		OTα	0.135	0	0	nd	
Turkey	233 (healthy individuals)	OTA	0.018	193	83	NA	[117]
Spain	72	OTA	0.112	9	12.5	0.057–0.562	[119]
		OTα	0.076	43	60.6	0.056–2.894	
Spain	27 (healthy volunteers)	OTA	1.5	3	11.1	uq	[120]
Italy	10 (5 female, 5 male)	OTA	0.02	9	90	max 0.25	[122]
Portugal	50 (female, winter)	OTA	0.008	43	86	max. 0.062	[123]
	45 (male, winter)	OTA	0.008	40	88.9	max. 0.071	
	50 (female, summer)	OTA	0.008	42	84	max. 0.040	
	45 (male, summer)	OTA	0.008	35	77.8	max. 0.039	
Belgium	40	OTA	0.06	4	10	0.1–0.61	[125]
		OTα	0.06	3	7.5	5.1–15	
		4-OH-OTA	0.24	2	2.5	uq	
Nigeria	120 (children, adolescents, adults)	OTA	0.15	34	28.3	max. 0.06	[98]
Germany	17 (mill workers)	OTA	0.02	17	100	0.016–0.228	[132]
		OTα	0.02	7	41	max. 0.31	
Germany	10 (infants)	OTA	0.05	8	80	max. 0.22	[133]
	11 (adults)	OTA	0.05	9	81.8	max. 0.319	
Turkey	28 (infants)	OTA	0.05	14	50	max. 1.36	[133]
Germany	50	OTA	0.02	50	100	0.02–1.82	[135]
		OTα	0.02	39	78	max. 14.25	
Nigeria	120	OTA	0.001	94	78.3	0.003–0.31	[136]
China	260 (adults)	OTA	0.1	3	1.2	0.153–0.557	[139]
		OTα	0.2	0	0	nd	
Czech Republic	50 (patients with KC)	OTA	0.002	31	62	0.001–0.0278	[140]
Portugal	94 (24 h urine)	OTA	0.02	16	17	max. 1.23	[91]
		OTα	0.02	0	0	nd	
France	9 (working exposure)	OTA	0.01	9	100	0.02–0.042	[147]
		OTα	0.25	0	0	nd	
Hungary	41 (healthy people)	OTA	0.001	41	100	0.071–1.024	[148]
	19 (coeliac patients)	OTA	0.001	19	100	0.052–0.243	
Qatar	559	OTA	1.5	0	0	nd	[149]
		OTB	5	3	0.5	uq	
Lebanon	43	OTA	0.01	43	100	0.007–0.058	[151]
Chile	172	OTA	2.1	1	0.6	uq	[152]
		OTα	NA	0	0	nd	
Zimbabwe	50	OTA	0.012	19	38	max. 0.11	[153]
Sweden	91 (adolescents)	OTA	0.013	74	81.3	max. 0.434	[95]
		OTα	0.14	0	0	nd	
		2′R-OTA	0.016	0	0	nd	
Spain	56 (healthy people)	OTA	10	0	0	nd	[87]
		OTB	10	5	9	max. 38.8	
United States of America	200 (pregnant women)	OTA	0.04	4	2	0.02–0.08	[170]
Brazil	26 (preschoolers)	OTA	0.06	10	38	NA	[157]
	29 (schoolers)	OTA	0.06	7	24	NA	
	49 (adolescents)	OTA	0.06	9	18	NA	
Spain	25	OTA	0.05	12	48	max. 0.258	[96]
Bangladesh	50 (pregnant women)	OTA	0.008	49	98	max. 2.37	[94]
		2’R-OTA	0.008	0	0	nd	
		OTα	0.12	4	8	uq	
		10-OH-OTA	0.02	0	0	nd	
		OTB-NAC	0.04	0	0	nd	
Nigeria	286 (adults—rural areas)	OTA	0.001	245	85.7	0.02–2.73	[159]

KC: kidney cancer; KIN: karyomegalic interstitial nephritis; LOQ: limit of quantification; NA: not available; nd: not detected; uq: unquantifiable (higher than the limit of detection, but below the limit of quantification). * Abbreviations defined in the footnote of Table 1.

**Table 4:** Selected studies on biomarkers of zearalenone in human urine (presented in ascending order by publication date).

Country	Sample Size (Particularities)	Biomarker *	LOQ (ng/mL)	No. Positive Samples	% Positive Samples	Range (ng/mL)	Ref.
Spain	27 (healthy volunteers)	ZEA	10	0	0	nd	[120]
Italy	10 (5 female, 5 male)	α-ZEL	1.6	0	0	nd	[122]
		β-ZEL	4.4	0	0	nd	
Belgium	40	ZEA	2.48	4	10	max. 12.6	[125]
		α-ZEL	1.22	0	0	nd	
		β-ZEL	2.2	4	10	4–24.8	
		ZEA-14-GlcA	7.3	0	0	nd	
Nigeria	120 (children, adolescents, adults)	ZEA	0.6	1	0.8	uq	[98]
		ZEA-14-GlcA	1	8	6.7	max. 44.5	
		α-ZEL	1	0	0	nd	
		β-ZEL	1	0	0	nd	
Germany	17 (mill workers)	ZEA	0.005	17	100	max. 0.1	[132]
		α-ZEL	0.025	4	23.5	max. 0.04	
		β-ZEL	0.025	3	17.6	max. 0.037	
United States of America	30 (pregnant subjects)	ZEA	NA	NA	NA	max. 0.31	[90]
		α-ZEL	NA	NA	NA	max. 0.25	
Nigeria	120	ZEA	0.003	98	81.7	0.03–19.99	[136]
		α-ZEL	0.01	5	4.2	0.52–2.52	
		β-ZEL	0.003	7	5.8	0.06–2.74	
China	301 (healthy volunteers aged 0–84 years)	ZEA	0.02	185	61.4	max. 3.7	[137]
		ZAN	0.03	2	0.67	max. 0.52	
		α-ZEL	0.04	3	1	max. 2.6	
		β-ZEL	0.06	19	6.3	max.	
		α-ZAL	0.04	0	0	nd	
		β-ZAL	0.02	0	0	nd	
China	260 (adults)	ZEA	0.1	18	6.9	0.056 -0.311	[139]
		ZAN	0.1	20	7.7	0.106–1.82	
		α-ZEL	0.5	0	0	nd	
		β-ZEL	0.5	0	0	nd	
		α-ZAL	0.2	0	0	nd	
		β-ZAL	0.5	0	0	nd	
		ZEA-14-GlcA	0.2	0	0	nd	
		ZAN-14-GlcA	0.1	0	0	nd	
Portugal	94 (24 h urine)	ZEA	0.8	24	26	max. 3.98	[91]
		ZAN	0.32	0	0	nd	
		α-ZEL	1.4	0	0	nd	
		β-ZEL	2.1	0	0	nd	
		α-ZAL	3.8	0	0	nd	
		β-ZAL	4.2	0	0	nd	
		α-ZEL-GlcA	3	0	0	nd	
		β-ZEL-GlcA	3.5	0	0	nd	
		ZEA-14-sulfate	4.9	0	0	nd	
		ZEA-14-GlcA	1.3	15	16	max. 25.7	
China	199 (children, adolescents, adults, elders)	ZEA	NA	175	87.9	0.01–3.77	[143]
		ZAN	NA	0	0	nd	
		α-ZEL	NA	51	25.6	0.02–2.66	
		β-ZEL	NA	48	24.1	0.03–2.84	
		α-ZAL	NA	0	0	nd	
		β-ZAL	NA	0	0	nd	
Iran	10 (healthy volunteers)	ZEA	6	0	0	nd	[146]
		ZAN	8	0	0	nd	
	17 (patients with EC)	ZEA	6	0	0	nd	
		ZAN	8	0	0	nd	
France	9 (working exposure)	ZEA	0.025	6	66.7	max. 0.364	[147]
		α-ZEL	1	2	11.1	max. 1.2	
		β-ZEL	0.5	0	0	nd	
Hungary	41 (healthy people)	ZEA	0.003	40	97.6	0.012–0.174	[148]
		α-ZEL	0.007	34	82.9	0.004–0.063	
		β-ZEL	0.025	8	19.5	0.013–0.033	
	19 (coeliac patients)	ZEA	0.003	19	100	0.011–0.121	
		α-ZEL	0.007	11	57.9	0.004–0.071	
		β-ZEL	0.025	2	10.5	max. 0.013	
Qatar	559	α-ZEL	5	22	3.9	5.08–7.01	[149]
		β-ZEL	5	42	7.5	5.11–19.58	
Chile	172	ZEA	1.7	1	0.6	uq	[152]
		α-ZEL	3.7	14	8	max. 16.5	
		β-ZEL	2.3	8	4.5	max. 31.3	
Zimbabwe	50	ZEA	0.035	5	10	max. 0.4	[153]
		α-ZEL	0.15	1	2	uq	
		β-ZEL	0.15	0	0	nd	
Sweden	91 (adolescents)	ZEA	0.57	0	0	nd	[95]
		ZAN	0.31	0	0	nd	
		α-ZEL	0.58	0	0	nd	
		β-ZEL	0.85	0	0	nd	
Spain	56 (healthy people)	ZEA	5	0	0	nd	[87]
		α-ZEL	5	0	0	nd	
United States of America	200 (pregnant women)	ZEA	0.1	126	63	0.07–2.5	[170]
		α-ZEL	0.2	44	22	0.1–0.9	
		β-ZEL	0.1	3	1.5	0.07–0.1	
Brazil	26 (preschoolers)	ZEA	0.05	15	57	NA	[157]
		α-ZEL	0.15	10	38	NA	
		β-ZEL	0.15	7	26	NA	
	29 (schoolers)	ZEA	0.05	19	66	NA	
		α-ZEL	0.15	8	28	NA	
		β-ZEL	0.15	4	14	NA	
	49 (adolescents)	ZEA	0.05	26	53	NA	
		α-ZEL	0.15	22	45	NA	
		β-ZEL	0.15	13	27	NA	
Spain	25	ZEA	0.01	16	64	max. 0.094	[96]
		ZAN	0.01	8	32	max. 0.099	
Bangladesh	50 (pregnant women)	ZEA	0.027	1	2	uq	[94]
		ZEA-14-GlcA	0.67	0	0	nd	
		α-ZEL	0.107	0	0	nd	
		β-ZEL	0.117	0	0	nd	
		α-ZEL-14-GlcA	1.33	0	0	nd	
		β-ZEL-14-GlcA	2.67	0	0	nd	
		ZAN-14-GlcA	2.67	1	2	max. 9.43	
Nigeria	286 (adults—rural areas)	ZEA	0.05	166	58	27.2–398	[159]
		α-ZEL	0.01	7	2.5	1.29–38.2	
		β-ZEL	0.01	4	1.4	0.96–1.87	
Bangladesh	446 (pregnant women)	ZEA	0.015	5	1.1	max. 0.031	[160]
		ZAN	0.039	0	0	nd	
		α-ZAL	0.029	0	0	nd	
		β-ZAL	0.035	0	0	nd	
		α-ZEL	0.013	0	0	nd	
		β-ZEL	0.035	0	0	nd	
		ZEA-14-GlcA	0.74	0	0	nd	
		ZEA-14-sulfate	0.011	0	0	nd	

EC: esophageal cancer; LOQ: limit of quantification; NA: not available; nd: not detected; uq: unquantifiable (higher than the limit of detection, but below the limit of quantification). * Abbreviations defined in the footnote of Table 1.

**Table 5:** Selected studies on biomarkers of deoxynivalenol in human urine (presented in ascending order by publication date).

Country	Sample Size (Particularities)	Biomarker *	LOQ (ng/mL)	No. Positive Samples	% Positive Samples	Range (ng/mL)	Ref.
China	11 (high-risk area)	DON	4	11	100	14–94	[102]
	4 (low-risk area)	DON	4	4	100	4–18	
UK	300	DON	0.6	296	98.7	NA	[107]
UK	210 (normal diet)	DON	NA	198	94.2	max. 78.2	[118]
	98 (partial cereal-based diet)	DON	NA	94	95.9	max. 34	
	40 (cereal-based diet)	DON	NA	17	42.5	max. 3.2	
Spain	27 (healthy volunteers)	DON	35	9	33.3	uq	[120]
Austria	4 (cereal-restricted diet)	DON-3-GlcA	10	0	0	nd	[121]
	4 (normal diet)	DON-3-GlcA	10	2	50	31–32	
Italy	10 (5 female, 5 male)	DON	1.6	7	70	1.1–14.2	[122]
		DOM-1	1.6	0	0	nd	
Belgium	40	DON	5.7	5	12.5	5.9–68.3	[125]
		DOM-1	1.3	0	0	nd	
		DON-3-GlcA	4.5	0	0	nd	
Nigeria	120 (children, adolescents, adults)	DON	4	1	0.8	uq	[98]
		DOM-1	30	0	0	nd	
		DON-3-GlcA	6	6	5	max. 8	
Germany	17 (mill workers)	DON	0.3	17	100	0.85–13.8	[132]
		DOM-1	0.2	10	58.8	max. 0.22	
UK	64 (vegetarians)	DON	NA	50	78.1	0.9–58.8	[134]
		DOM-1	NA	0	0	nd	
	62 (normal diet)	DON	NA	62	100	max. 60.5	
		DOM-1	NA	0	0	nd	
Nigeria	120	DON	0.15	23	19.2	0.08–6.22	[136]
UK	40 (children)	DON	0.25	NA	NA	1.2–140.9	[138]
		DOM-1	0.5	0	0	nd	
	39 (adolescents)	DON	0.25	NA	NA	max. 104.3	
		DOM-1	0.5	0	0	nd	
China	260 (adults)	DON	1	26	10	1.39–14.7	[139]
		3-AcDON	1	0	0	nd	
		15-AcDON	1	0	0	nd	
		DON-3-GlcA	1	41	15.8	0.583–5.84	
		DON-15-GlcA	NA	114	43.8	0.828–37.7	
Portugal	94 (24 h urine)	DON	1	59	63	max. 36.31	[91]
		DOM-1	0.5	37	39	max. 5.13	
		3-AcDON	0.8	0	0	nd	
		15-AcDON	0.8	0	0	nd	
		DON-3G	1.1	7	7	max. 2.09	
		DON-3-GlcA	1	35	37	max. 34.67	
		DON-15-GlcA	0.9	48	51	max. 204.17	
Italy	406	DON	0.5	151	37.2	mean 7.80	[141]
		DOM-1	0.51	6	1.5	NA	
Bangladesh	120 (children and infants)	DON	0.3	40	33.3	0.3–8.6	[142]
		DOM-1	0.2	0	0	nd	
Pakistan	264	DON	0.5	54	20	max. 1.247	[145]
Iran	10 (healthy volunteers)	DON	0.25	1	10	8.42	[146]
		3-AcDON	0.5	0	0	nd	
		15-AcDON	0.5	0	0	nd	
	17 (patients with EC)	DON	0.25	0	0	nd	
		3-AcDON	0.5	0	0	nd	
		15-AcDON	0.5	0	0	nd	
France	9 (working exposure)	DON	0.05	9	100	3.9–18.8	[147]
Hungary	41 (healthy people)	DON	0.42	18	43.9	2.01–18.947	[148]
		DOM-1	0.45	0	0	nd	
	19 (coeliac patients)	DON	0.42	9	47.3	3.876–9.484	
		DOM-1	0.45	0	0	nd	
Norway	257 (normal diet and vegetarians)	DON	0.015	256	99.6	NA	[150]
		DOM-1	0.27	31	12	NA	
Chile	172	DON	20.1	124	73	max. 61.1	[152]
		DOM-1	NA	0	0	nd	
Sweden	91 (adolescents)	DON	3.03	69	75.8	max. 136	[95]
Germany	360 (24-h urine samples)	DON	0.3	357	99	0.3–99.1	[154]
France	300 (first sample morning)	DON	0.05	280	93.3	max. 108.1	[155]
Germany	120 (24 h)	DON	0.3	118	98.3	max. 24.9	[155]
Iceland	171 (spot sample)	DON	0.14	160	93.6	max. 33.45	[155]
Luxembourg	191 (spot sample)	DON	0.5	183	95.8	max. 124	[155]
Poland	193 (spot sample)	DON	0.5	192	99.5	max. 133.2	[155]
Portugal	295 (first sample morning)	DON	0.05	288	97.6	max. 77.87	[155]
USA	200 (pregnant women)	DON	1.9	198	99	0.95–436	[170]
		DOM-1	0.94	5	2.5	0.5–2.6	
Brazil	26 (preschoolers)	DON	1.32	4	15	NA	[157]
	29 (schoolers)	DON	1.32	6	21	NA	
	49 (adolescents)	DON	1.32	5	10	NA	
Bangladesh	50 (pregnant women)	DON	0.67	2	4	max. 1.05	[94]
		DON-3-GlcA	1.3	6	12	max. 11	
Nigeria	286 (adults—rural areas)	DON	0.5	15	5.2	4.13–47.2	[159]

EC: esophageal cancer; LOQ: limit of quantification; NA: not available; nd: not detected; uq: unquantifiable (higher than the limit of detection, but below the limit of quantification). * Abbreviations defined in the footnote of Table 1.

**Table 6:** Selected studies on biomarkers of T-2 and HT-2 toxins in human urine (presented in ascending order by publication date).

Country	Sample Size (Particularities)	Biomarker *	LOQ (ng/mL)	No. Positive Samples	% Positive Samples	Range (ng/mL)	Ref.
Spain	27 (healthy volunteers)	T-2	6	0	0	nd	[120]
		HT-2	10	0	0	nd	
Belgium	40	T-2	0.1	0	0	nd	[125]
		HT-2	0.84	0	0	nd	
Nigeria	120 (children, adolescents, adults)	T-2	1	0	0	nd	[98]
		HT-2	40	0	0	nd	
China	260 (adults)	T-2	0.1	6	2.3	0.248–3.61	[139]
		HT-2	0.5	0	0	nd	
Portugal	94 (24 h urine)	T-2	0.2	0	0	nd	[91]
		HT-2	0.4	0	0	nd	
Iran	10 (healthy volunteers)	T-2	1	0	0	nd	[146]
		HT-2	2	1	10	23.97	
	17 (patients with EC)	T-2	1	1	5.8	50.09	
		HT-2	2	3	17.6	18.91–50.38	
France	9 (working exposure)	T-2	0.5	0	0	nd	[147]
		HT-2	1	0	0	nd	
Hungary	41 (healthy people)	T-2	0.83	0	0	nd	[148]
		HT-2	20.8	0	0	nd	
	19 (coeliac patients)	T-2	0.83	0	0	nd	
		HT-2	20.8	0	0	nd	
Qatar	559	T-2	1.5	3	0.5	uq	[149]
Sweden	91 (adolescents)	T-2	0.33	0	0	nd	[95]
		HT-2	4.5	0	0	nd	
Brazil	26 (preschoolers) 29 (schoolers) 49 (adolescents)	T-2	0.04	3	11	NA	[157]
		HT-2	0.08	1	4	uq	
		T-2	0.04	2	7	NA	
		HT-2	0.08	0	0	nd	
		T-2	0.04	2	4	NA	
		HT-2	0.08	1	2	uq	
Norway	117 (adults)	HT-2	0.05	99	85	0.078–0.476	[158]
Bangladesh	50 (pregnant women)	T-2	0.08	5	10	uq	[94]
		HT-2	5	0	0	nd	
		HT-2-3-GlcA	0.02	0	0	nd	
		HT-2-4-GlcA	0.016	0	0	nd	

EC: esophageal cancer; LOQ: limit of quantification; NA: not available; nd: not detected; uq: unquantifiable (higher than the limit of detection, but below the limit of quantification). * Abbreviations defined in the footnote of Table 1.

**Table 7:** Selected studies on biomarkers of fumonisins in human urine (presented in ascending order by publication date).

Country		Sample Size (Particularities)	Biomarker *	LOQ (ng/mL)	No. Positive Samples	% Positive Samples	Range (ng/mL)	Ref.
Mexic		75 (only women)	FB1	NA	56	75	0.02–9.312	[111]
Portugal		68 (urban & rural areas)	FB1	10	0	0	nd	[115]
			FB2	10	0	0	nd	
Korea		12	FB1	0.022	0	0	nd	[116]
			FB2	0.01	0	0	nd	
Spain		27 (healthy volunteers)	FB1	15	0	0	nd	[120]
			FB2	15	0	0	nd	
Belgium		40	FB1	0.1	0	0	nd	[125]
			HFB1	1.02	0	0	nd	
Nigeria		120 (children, adolescents, adults)	FB1	2	16	13.3	max. 12.8	[98]
			FB2	0.7	2	1.7	max. 1	
Nigeria		120	FB1	0.01	85	70.8	0.08–14.88	[136]
China		260 (adults)	FB1	0.5	8	3.1	0.230–1.33	[139]
Portugal		94 (24 h urine)	FB1	0.42	2	2	max. 0.48	[91]
			FB2	0.36	0	0	nd	
			FB3	0.37	0	0	nd	
			HFB1	0.42	0	0	nd	
France		9 (working exposure)	FB1	0.25	0	0	nd	[147]
Hungary		41 (healthy people)	FB1	0.017	41	100	0.141–1.525	[148]
			FB2	0.016	41	100	0.008–0.875	
		19 (coeliac patients)	FB1	0.017	19	100	0.146–0.356	
			FB2	0.016	19	100	0.016–0.226	
Qatar		559	FB1	0.2	1	0.2	uq	[149]
Zimbabwe		50	FB1	0.014	50	100	0.032–4.6	[153]
Sweden		91 (adolescents)	FB1	0.53	0	0	nd	[95]
Brazil		26 (preschoolers)	FB1	0.05	9	34	NA	[157]
			FB2	0.06	4	15	NA	
		29 (schoolers)	FB1	0.05	14	48	NA	
			FB2	0.06	2	7	NA	
		49 (adolescents)	FB1	0.05	14	29	NA	
			FB2	0.06	7	14	NA	
Bangladesh		50 (pregnant women)	FB1	0.005	0	0	nd	[94]
			FB2	0.01	0	0	nd	
Nigeria	286 (adults—rural areas)		FB1	0.01	204	71.3	0.78–566	[159]

LOQ: limit of quantification; NA: not available; nd: not detected; uq: unquantifiable (higher than the limit of detection, but below the limit of quantification). * Abbreviations defined in the footnote of Table 1.

**Table 8:** Selected studies on biomarkers of patulin in human urine.

Country	Sample Size (Particularities)	Biomarker *	LOQ (ng/mL)	No. Positive Samples	% Positive Samples	Range (ng/mL)	Ref.
Portugal	94 (24 h urine)	PAT	1	0	0	nd	[91]
Tunisia	50 (healthy volunteers)	PAT	2.65	0	0	nd	[144]
	50 (CRC patients)	PAT	2.65	0	0	nd	

CRC: colorectal cancer; LOQ: limit of quantification; nd: not detected. * Abbreviations defined in the footnote of Table 1.

**Table 9:** Selected studies on biomarkers of citrinin in human urine (presented in ascending order by publication date).

Country	Sample Size (Particularities)	Biomarker *	LOQ (ng/mL)	No. Positive Samples	% Positive Samples	Range (ng/mL)	Ref.
Belgium	40	CIT	5.76	1	2.5	6.8	[125]
Turkey	10 (adults and infants)	CIT	0.05	8	80	0.16–0.79	[126]
		DH-CIT	0.1	5	50	0.15–1.12	
Germany	50 (healthy adults)	CIT	0.05	41	82	max 0.08	[129]
		DH-CIT	0.1	42	84	max. 0.51	
Germany	17 (mill workers)	CIT	0.05	17	100	max. 0.076	[132]
		DH-CIT	0.1	17	100	max. 0.506	
Nigeria	120	CIT	0.01	79	65.8	0.015–241.46	[136]
		DH-CIT	0.01	69	57.5	0.05–16.89	
Czech Republic	50 (patients with KC)	CIT	0.05	45	90	max. 0.087	[140]
		DH-CIT	0.1	50	100	max. 0.160	
Portugal	94 (24 h urine)	CIT	1	1	1	1.2	[91]
Tunisia	50 (healthy volunteers)	CIT	0.2	36	72	max. 5.72	[144]
	50 (CRC patients)	CIT	0.2	38	76	max. 0.96	
Qatar	559	CIT	1.5	6	1.1	uq	[149]
Zimbabwe	50	CIT	0.32	7	14	max. 2.3	[153]
		DH-CIT	0.23	9	18	max. 1.3	
Sweeden	91 (adolescents)	CIT	0.2	0	0	nd	[95]
		DH-CIT	0.06	50	54.9	max. 0.97	
USA	200 (pregnant women)	DH-CIT	0.6	4	2	0.82–66	[170]
Spain	25	CIT	0.5	0	0	nd	[96]
Bangladesh	50 (pregnant women)	CIT DH-CIT	0.125 0.016	20 39	40 78	max. 4.62 max. 6.11	[94]
Nigeria	286 (adults—rural areas)	CIT DH-CIT	0.05 0.05	107 172	37.4 60.1	5.89–425 0.53–190	[159]

CRC: colorectal cancer; KC: kidney cancer; LOQ: limit of quantification; nd: not detected; uq: unquantifiable (higher than the limit of detection, but below the limit of quantification). * Abbreviations are defined in the footnote of Table 1.

**Table 10:** Selected studies on biomarkers of emerging mycotoxins in human urine (presented in ascending order by publication date).

Country	Sample Size (Particularities)	Biomarker *	LOQ (ng/mL)	No. Positive Samples	% Positive Samples	Range (ng/mL)	Ref.
Nigeria	120 (children, adolescents, adults)	NIV	4	0	0	nd	[98]
Nigeria	120	NIV	0.1	40	33.3	0.24–3.02	[136]
China	260 (adults)	FUS-X	1	0	0	nd	[139]
Portugal	94 (24 h urine)	NIV	0.4	0	0	nd	[91]
		DAS	1.5	0	0	nd	
		NEO	1.2	0	0	nd	
		FUS-X	0.8	0	0	nd	
		STG	0.05	0	0	nd	
Iran	10 (healthy volunteers)	FUS-X	4	0	0	nd	[146]
		NIV	1	0	0	nd	
		NEO	0.5	4	40	10.5–22.5	
	17 (patients with EC)	FUS-X	4	0	0	nd	
		NIV	1	0	0	nd	
		NEO	0.5	1	5.8	12.9	
Hungary	41 (healthy people)	NIV	0.06	1	2.4	0.475	[148]
	19 (coeliac patients)	NIV	0.06	0	0	nd	
Qatar	559	STG	0.2	5	0.9	uq	[149]
Spain	25	AME	0.5	0	0	nd	[96]
		AOH	0.5	17	68	max. 8.683	
		STG	0.005		48	max. 0.03	
Bangladesh	50 (pregnant women)	STG	0.002	38	76	max. 0.033	[94]
		ALT	2	0	0	nd	
		AME	0.004	7	14	0.009	
		AOH	0.132	1	2	uq	
		TeA + allo-TeA	0.192	43	86	max. 7.69	
		CPA	0.008	0	0	nd	
		PENA	0.027	0	0	nd	
		PAX	0.17	0	0	nd	
		BEA	0.067	0	0	nd	
		ENA	0.012	0	0	nd	
		ENA1	0.012	4	8	max. 0.034	
		ENB	0.002	2	4	max. 0.005	
		ENB1	0.01	0	0	nd	
Nigeria	286 (adults—rural areas)	NIV	0.1	39	13.6	1.24–13.8	[159]
Bangladesh	446 (pregnant women)	AME	0.01	153	34	max. 0.54	[160]
		AOH	0.06	40	8.9	max. 0.47	

EC: esophageal cancer; LOQ: limit of quantification; nd: not detected; uq: unquantifiable (higher than the limit of detection, but below the limit of quantification). * Abbreviations are defined in the footnote of Table 1.

## Data Availability

No new data were created or analyzed in this study.

## Abbreviations

The following abbreviations are used in this manuscript:

10-OH-OTA	10-Hydroxy-ochratoxin A
15-AcDON	15-Acetyl-deoxynivalenol
15-AcDON-3-GlcA	15-Acetyl-deoxynivalenol-3-glucuronide
2′R-OTA	2′R-Ochratoxin A
3-AcDON	3-Acetyl-deoxynivalenol
3-AcDON-15-GlcA	3-Acetyl-deoxynivalenol-15-glucuronide
4-OH-OTA	4-Hydroxy-ochratoxin A
ADI	Acceptable daily intake
ADME	Absorption, distribution, metabolism, and excretion
AFB1	Aflatoxin B1
AFB1-N7-Gua	Aflatoxin B1-N7-guanine
AFB2	Aflatoxin B2
AFG1	Aflatoxin G1
AFG2	Aflatoxin G2
AFL	Aflatoxicol
AFM1	Aflatoxin M1
AFM2	Aflatoxin M2
AFs	Aflatoxins
ALARA	As low as reasonably achievable
ALT	Altenuene
AME	Alternariol monomethyl ether
AOH	Alternariol
BEA	Beauvericin
BMDL10	Benchmark Dose Lower Confidence Limit 10%
bw	Body weight
CAC	Codex Alimentarius Commission
CIT	Citrinin
CPA	Cyclopiazonic acid
DAS	Diacetoxyscirpenol
DH-CIT	Dihydrocitrinone
DLLME	Dispersive liquid–liquid microextraction
d-SPE	Dispersive solid-phase extraction
DOM-1	Deepoxy-deoxynivalenol
DOM-GlcA	Deepoxy-deoxynivalenol-glucuronides
DON	Deoxynivalenol
DON-15-GlcA	Deoxynivalenol-15-O-β-glucuronide
DON-3G	Deoxynivalenol-3-glucoside
DON-3-GlcA	Deoxynivalenol-3-O-β-glucuronide
DON-GlcA	Deoxynivalenol-glucuronides
EDI	Estimated daily intake
EFSA	European Food Safety Authority
ELISA	Enzyme-linked immunosorbent assay
ENA	Enniatin A
ENA1	Enniatin A1
ENB	Enniatin B
ENB1	Enniatin B1
ENs	Enniatins
FAO	Food and Agriculture Organization
FB1	Fumonisin B1
FB2	Fumonisin B2
FB3	Fumonisin B3
FDA	Food and Drug Administration
FLD	Fluorescence detection
FUMOs	Fumonisins
FUS-X	Fusarenone-X
GC	Gas chromatography
GC–MS/MS	Gas chromatography–tandem mass spectrometry
GC–QqQ–MS/MS	Gas chromatography–triple quadrupole–tandem mass spectrometry
HBM	Human biomonitoring
HBM4EU	European Human Biomonitoring Initiative
HFB1	Hydrolyzed fumonisin B1
HLB	Hydrophilic–lipophilic balanced
HPLC	High-performance liquid chromatography
HPLC-ESI-MS	High-performance liquid chromatography–electrospray ionization–single quadrupole mass spectrometry
HPLC-FLD	High-performance liquid chromatography–fluorescence detection
HPLC-MS	High-performance liquid chromatography–mass spectrometry
HPLC-MWCNTs	High-performance liquid chromatography–multi-wall carbon nanotubes detection
HQ	Hazard Quotient
HRMS	High-resolution mass spectrometry
HT-2	HT-2 toxin
HT-2-3-GlcA	HT-2 toxin-3-glucuronide
HT-2-4-GlcA	HT-2 toxin-4-glucuronide
IAC	Immunoaffinity column
IARC	International Agency for Research on Cancer
JECFA	Joint FAO/WHO Expert Committee on Food Additives
LB	Lower bound
LC	Liquid chromatography
LC-ESI-QTOF-MS	Liquid chromatography coupled to quadrupole time-of-flight mass spectrometry with electrospray ionization
LC-FLD	Liquid chromatography–fluorescence detection
LC-HRMS	Liquid chromatography–high-resolution mass spectrometry
LC-MS/MS	Liquid chromatography–tandem mass spectrometry
LLE	Liquid–liquid extraction
LOD	Limit of detection
LOQ	Limit of quantification
ML	Maximum level
MOE	Margin of exposure
MON	Moniliformin
MOS	Margin of safety
MPL	Maximum permitted level
MS	Mass spectrometry
MS/MS	Tandem mass spectrometry
MWCNTs	Multi-wall carbon nanotubes
NEO	Neosolaniol
NIV	Nivalenol
NOAEL	No-observed-adverse-effect level
NRC	National Research Council
OTA	Ochratoxin A
OTA-8-GlcA	Ochratoxin A-8-β-glucuronide
OTB	Ochratoxin B
OTC	Ochratoxin C
OTB-NAC	Ochratoxin-N-acetyl-L-cysteine
OTα	Ochratoxin α
OTβ	Ochratoxin β
PAT	Patulin
PAX	Paxilline
PDI	Probable daily intake
PENA	Penitrem A
PMTDI	Provisional maximum tolerable daily intake
QuEChERS	Quick, Easy, Cheap, Effective, Rugged, and Safe
RASFF	Rapid Alert System for Food and Feed
rpm	Revolutions per minute
SALLE	Salting-out-assisted liquid–liquid extraction
SLE	Supported liquid extraction
SPE	Solid-phase extraction
SPME	Solid-phase microextraction
STG	Sterigmatocystin
T-2	T-2 toxin
TeA	Tenuazonic acid
TDI	Tolerable daily intake
UB	Upper bound
UHPLC	Ultra-high-performance liquid chromatography
UHPLC-MS/MS	Ultra-high-performance liquid chromatography–tandem mass spectrometry
USA	United States of America
WHO	World Health Organization
ZAN	Zearalanone
ZAN-14-GlcA	Zearalanone-14-glucuronide
ZEA	Zearalenone
ZEA-14-GlcA	Zearalenone-14-O-β-glucuronide
ZEA-14-sulfate	Zearalenone-14-sulfate
α-ZAL	α-Zearalanol
α-ZEL	α-Zearalenol
α-ZEL-GlcA	α-Zearalenol-glucuronides
β-ZAL	β-Zearalanol
β-ZEL	β-Zearalenol
β-ZEL-GlcA	β-Zearalenol-glucuronides
